# A motor association area in the depths of the central sulcus

**DOI:** 10.1038/s41593-023-01346-z

**Published:** 2023-05-18

**Authors:** Michael A. Jensen, Harvey Huang, Gabriela Ojeda Valencia, Bryan T. Klassen, Max A. van den Boom, Timothy J. Kaufmann, Gerwin Schalk, Peter Brunner, Gregory A. Worrell, Dora Hermes, Kai J. Miller

**Affiliations:** 1 Medical Scientist Training Program, Mayo Clinic, Rochester, MN USA; 2 Neurosurgery, Mayo Clinic, Rochester, MN USA; 3 Department of Physiology and Biomedical Engineering, Mayo Clinic, Rochester, MN USA; 4 Neurology, Mayo Clinic, Rochester, MN USA; 5 Radiology, Mayo Clinic, Rochester, MN USA; 6 Chen Frontier Lab for Applied Neurotechnology, Tianqiao and Chrissy Chen Institute, Shanghai, China; 7 Neurosurgery, Fudan University/Huashan Hospital, Shanghai, China; 8 Neurosurgery, Washington University School of Medicine, St Louis, MO USA

**Keywords:** Motor cortex, Sensorimotor processing, Motor control

## Abstract

Cells in the precentral gyrus directly send signals to the periphery to generate movement and are principally organized as a topological map of the body. We find that movement-induced electrophysiological responses from depth electrodes extend this map three-dimensionally throughout the gyrus. Unexpectedly, this organization is interrupted by a previously undescribed motor association area in the depths of the midlateral aspect of the central sulcus. This ‘Rolandic motor association’ (RMA) area is active during movements of different body parts from both sides of the body and may be important for coordinating complex behaviors.

## Abstract (web summary)

Using depth electrodes in human patients, scientists at the Mayo Clinic found that the map of the body in motor cortex extends deep into the central sulcus. Unexpectedly, the nonsomatotopic ‘Rolandic motor association’ (RMA) area interrupts this organization.

## Main

The organized representation of body movements on the posterior convexity of the precentral gyrus (PCG), named the homunculus, was discovered nearly a century ago by direct brain surface stimulation in awake neurosurgical patients^1^—it follows a medial-to-lateral pattern of lower extremities, upper extremities and face. Subsequent measurements of task-driven changes in functional magnetic resonance imaging (fMRI), magnetoencephalography^2^ and brain surface electrophysiology with electrocorticography (ECoG) have all recapitulated this somatotopic organization^3–5^. Neurons from each of these somatotopic areas in the PCG, called primary motor cortex, communicate with the brainstem and spinal cord to produce body movements. Primary brain areas are defined by having a simple chain of synaptic connections to the periphery and a direct topographical mapping to the outside world^6^—retinotopy in the calcarine cortex (visual), tonotopy in the transverse temporal gyrus (auditory), and somatotopy in the postcentral gyrus (sensation) and the PCG (movement)^7^. Brain areas that can be related to these functions but are not themselves primary are called association areas. Association areas may or may not exhibit topographic organization and are often found to coordinate basic topographic features for a more complex purpose^8^. Our research began as an effort to simply characterize the PCG primary motor cortex electrophysiologically throughout its three-dimensional (3D) volume, measuring from superficial and deep areas simultaneously with penetrating stereoelectroencephalographic (sEEG) depth electrodes placed in patients’ brains for clinical practice. We expected to find only classic primary motor properties along the anterior bank of the central sulcus as fMRI-based mapping has^4,9^, but instead found surprising evidence for an association area interrupting the otherwise somatotopic representation.

**Fig. 1:**
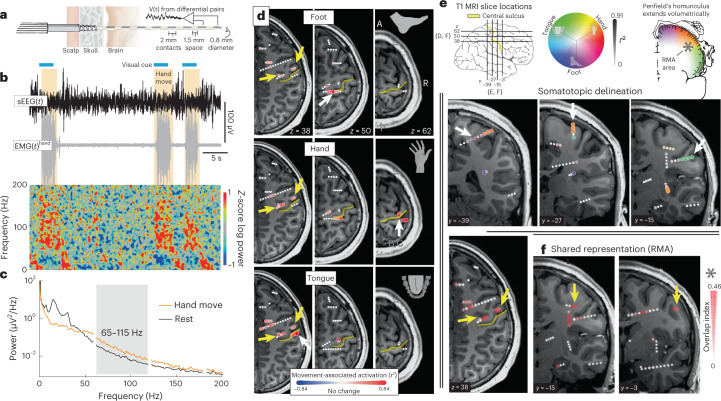
The sEEG signal and electrophysiologic changes during movement. **a**, Schematic of sEEG lead. **b**, sEEG bipolar pair voltage timeseries from site noted in midright panel of **d** and spectrogram aligned to EMG recorded from the forearm. The voltage timeseries shows event-locked raw voltage deflections, while the spectrogram exhibits characteristic broadband spectral increases (>50 Hz) and narrow band oscillatory decreases (10–30 Hz) during movement. **c**, Averaged power spectral density from timeseries in **b** shows characteristic changes. The 65–115 Hz range chosen to capture local brain activity is shown in gray (line noise and harmonics excluded at 60 Hz, 120 Hz and 180 Hz). **d**, Axial T1 MRI slices with overlaid neural activity maps of foot (top row), hand (middle) and tongue (bottom) movements (signed significant *r*^2^, *P* < 0.05 determined by a two-sample—move and rest—*t*-test, 65–115 Hz power). Each bipolar channel is projected to closest axial slice (<6 mm). White arrowheads indicate example sites of clear somatotopic specificity and yellow arrowheads indicate sites that are active during all movements. **e**, Coronal T1 MRI slices with overlaid somatotopic delineation maps. The circular colormap is generated by plotting the vector sum of individual foot, hand and tongue *r*^2^ values. Color specifies somatotopic tuning while the diameter and intensity indicate the magnitude of the vector sum. Note that a channel that is equally active during all three movement types will be plotted small and white. White arrowheads point to corresponding sites in **d**. **f**, Axial (leftmost) and coronal T1 MRI slices with overlaid maps of shared representation with scaled asterisks symbols (overlap quantified by geometric mean of significant—*P* < 0.05—hand, tongue and foot *r*^2^ values). Yellow arrowheads point to corresponding sites in **d**. All panels show data recorded in subject 1.

In our treatment of patients with drug-resistant focal epilepsy, sEEG depth electrodes may help to identify where seizures originate from and propagate to in the brain^10^. sEEG has largely replaced brain surface ECoG arrays in recent years^11^, as it is minimally traumatic, allows for volumetric characterization of seizure networks and is well tolerated^12,13^. As a complement to electrical stimulation mapping, which perturbs the brain to characterize function, we also analyze electrophysiological changes during simple behavioral tasks to map neural activity in the immediate vicinity of each electrode. The electrical potential signals measured by sEEG from cortex during behavior show the same general features as ECoG^14,15^, some of which are: event-locked raw voltage deflections, oscillations (rhythms) and broadband (power-law) spectral changes (Fig. 1). As in ECoG, we find that, in pericentral areas, simple movements produce (1) widespread decrease in power in narrow band oscillations in the ~10–30 Hz range and (2) focal broadband spectral increases above ~50 Hz that we capture between 65 Hz and 115 Hz (Fig. 1). Such broadband changes have been shown to be a general correlate of neural population firing rate^16^.

**Fig. 2:**
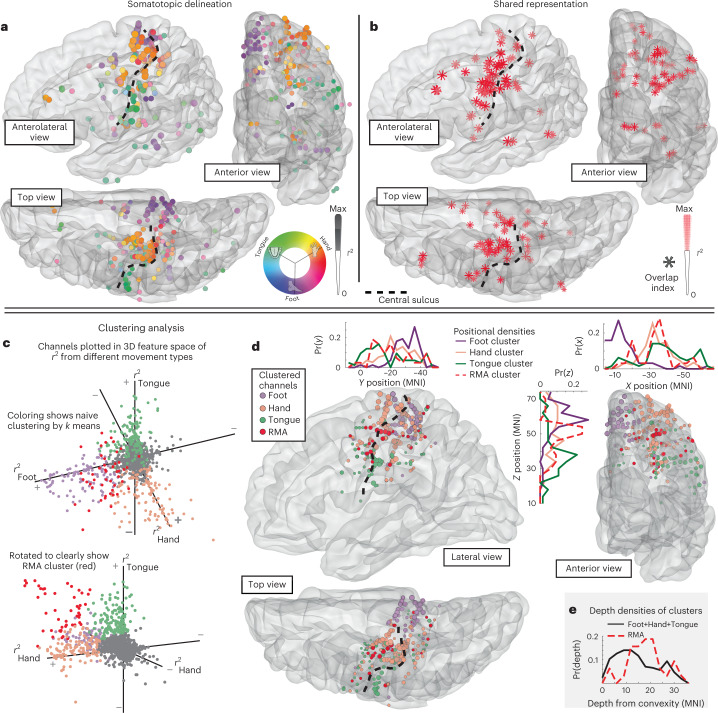
Somatotopic and shared representation across subjects in a common space. **a**, Each subject’s anatomy was warped to the left hemisphere of the MNI152 atlas, and somatotopic delineation is plotted in aggregate across subjects (only channels exceeding 50% of the within-subject maximum are included). **b**, Shared activity is plotted in aggregate with scaled asterisks symbols (again, only channels exceeding 50% of the within-subject maximum are included). **c**, A clustering analysis was performed to see what representations emerge from the data naïvely. *K*-means clustering was applied to a 3D feature space of foot, hand and tongue movement *r*^2^ values, and analyses found that five clusters best captured the tradeoff between error and overfitting. The emergent clusters are shown color-coded in the 3D *r*^2^ feature space and clearly correspond to sites representing hand, tongue or foot movement, sites of shared representation (RMA) and sites unrelated to movement (Supplementary Fig. 10). **d**, Channels within pericentral cortex are plotted with colors from **c**, omitting the cluster for sites unrelated to movement. Inset histograms show the density of clustered sites in *x*, *y* and *z* coordinates in MNI space. Note the position of the RMA cluster in the sulcal depths at the mid-lateral aspect of the PCG. **e**, A histogram of depths shows that RMA cluster sites are deeper in the brain than somatotopic sites. Note that while it appears that tongue-selective somatotopic sites are posterior to the most superficial aspect of the central sulcus on the MNI rendering, examination of individual axial MRI slices shows them to be mostly anterior to the sulcus (Extended Data Fig. 4).

**Fig. 3:**
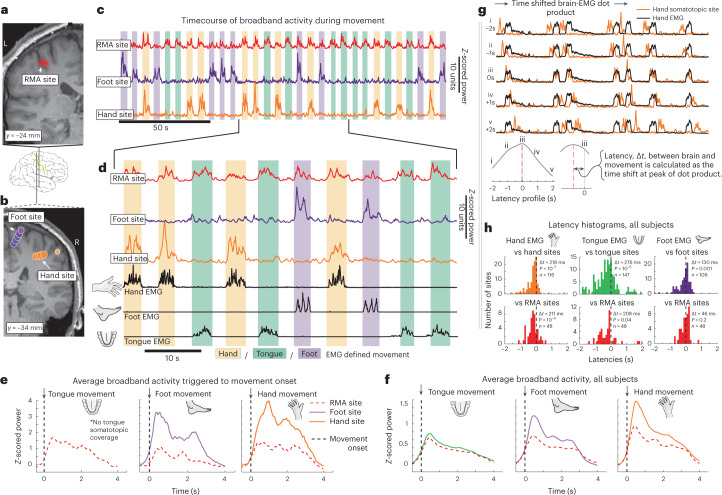
Temporal dynamics of neural activity in the PCG. **a**, Coronal T1 MRI slice through the PCG and central sulcus in the left hemisphere, with overlaid shared representation map, showing RMA area sites. **b**, Coronal T1 MRI slice with overlaid somatotopic delineation map. **c**, Timecourse of normalized broadband activity (65–115 Hz) for RMA, foot and hand channels (white arrowheads in **a** and **b**) for a full experimental run (30 trials). Background shading indicates EMG-defined movement periods of the hand (red), foot (purple) and tongue (green). **d**, Magnified inset from **c**, with EMG included. **e**, Brain activity averaged to onset of tongue, foot and hand EMG. This subject had no somatotopic tongue site to display. **f**, Brain activity averaged to onset of tongue, foot and hand EMG across all subjects. As each channel’s broadband is normalized to itself, the magnitudes of these averaged traces are not meaningful, but the shapes are. All traces were shifted to start at a *z*-scored power of 0 to allow for more natural comparison. **g**, The latency between brain and movement was calculated as the peak of a sliding dot product between sEEG broadband and rectified EMG. **h**, Histograms of latency between somatotopic hand/foot/tongue sites and their paired EMG traces, as well as RMA site to each EMG show that all brain sites lead EMG (*P* values determined by two-sided one-sample *t*-test versus 0). Note that there was no significant difference in latency when comparing somatotopic and RMA sites (*P* = 0.24, unpaired *t*-test of 369 somatotopic latencies versus 144 RMA latencies). Panels **a**–**e** show data recorded in subject 2.

**Extended Data Fig. 1:**
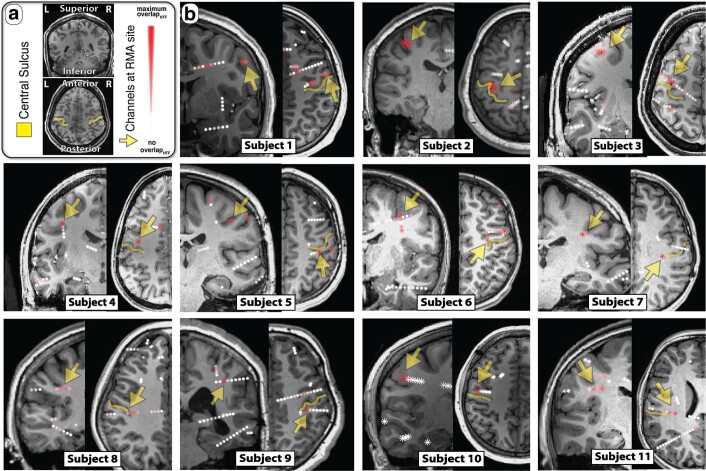
The Rolandic Motor Association area within the central sulcus. **(a)** Orientation of coronal and axial slices **(b)** Axial and coronal views demonstrate localization of channels with shared representation between hand, tongue, and foot movement within the central sulcus. We call this region of shared representation the Rolandic motor association (RMA) area (yellow arrow).

**Extended Data Fig. 2:**
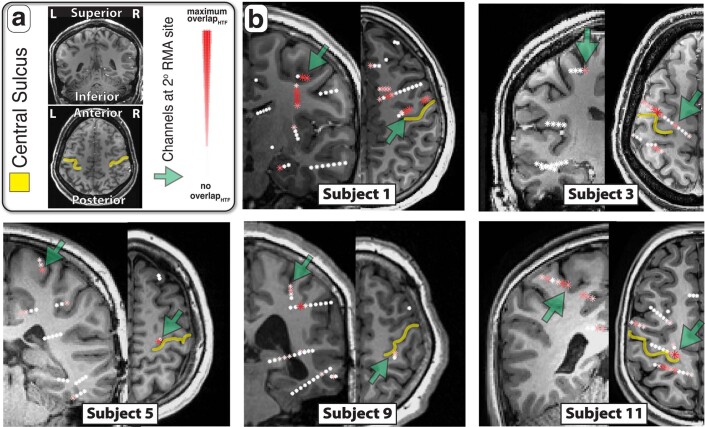
A secondary Rolandic Motor Association area within the central sulcus. **(a)** Orientation of coronal and axial slices **(b)** Axial and coronal views demonstrate localization of channels with shared representation between hand, tongue, and foot movement within the central sulcus. Green arrows indicate the channels which lie within the central sulcus at a secondary RMA site, superior and medial to the primary RMA site found in every subject.

**Extended Data Fig. 3:**
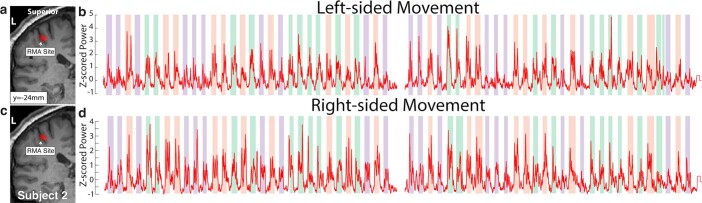
Rolandic Motor Association area activity for movements of both sides of the body. **(a)** Electrodes within RMA during right-sided movement of hand, tongue, foot **(b)** Time-series of broadband power across two consecutive runs from the RMA site during left-sided movement **(c)** Electrodes within RMA during right-sided movement of hand, tongue, foot **(d)** Time-series of broadband power across two consecutive runs from the RMA site during right-sided movement.

Subjects performed a simple block-designed task of randomly interleaved tongue, hand or foot movements (contralateral to SEEG array) with rest in between, while electromyography (EMG) was recorded from each body area. A simple analysis of broadband changes extended the classic somatotopic representation of individual body parts into the sulcal depths of the PCG, with foot along the midline, hand in the superior-lateral part and tongue in the lateral aspect^1^ (Figs. 1 and 2). Unexpectedly, the organized topology of the homunculus was interrupted by a region of shared representation in the depths of the central sulcus, at its midlateral aspect, that was electrophysiologically active during all three movement types. We call this the ‘Rolandic motor association’ (RMA) area in reference to the historical name of the central sulcus (fissure of Rolando)^17^. This RMA area was independently observed in all 13 subjects and was distinct from surrounding movement-specific somatotopic regions in each case (Figs. 1 and 2, Extended Data Figs. 1 and 2 and Supplementary Figs. 13 and 19–33). Activity in the RMA precedes muscle movement (Fig. 3 and Supplementary Figs. 16–18). In one patient (subject 2) who performed tasks bilaterally, the RMA area was active during both ipsilateral and contralateral body movements (Fig. 3 and Extended Data Fig. 3). Standard clinical stimulation mapping incidentally included the RMA for one patient (subject 2) and did not disrupt movement or speech function (up to 5 mA bipolar testing, although 2 mA stimulation at primary motor foot-specific regions produced muscle contraction).

Combined with recent fMRI studies^4^, our electrophysiological finding of somatotopic delineation throughout the volume of the pericentral brain is an important extension of Penfield’s classical homunculus (Fig. 2) (ref. ^1^). Our measurements also succinctly establish that this RMA area is a different phenomenon, lacking the somatotopically specific organization found in the homunculus. Because the RMA is not plainly related to any single movement function, we believe that it is likely an association area that helps to coordinate different effectors of movement.

Awake motor mapping with intraoperative stimulation and ECoG^1,18^ has likely overlooked this RMA area because of its relatively inaccessible location within the central sulcus (that is the depths of Brodmann area 4). However, a growing body of work has recently established that the somatotopic organization on the superficial convexity of the PCG (that is Brodmann area 6—BA6) is also interrupted by regions that have integrative roles to coordinate behavior. PCG area 55b, which lies superficial and anterior to the RMA area^19^, has recently been associated with both speech production^20^ and music rhythm attunement^21^. More dorsally, microelectrode recordings from the premotor (BA6) portion of the PCG hand area of tetraplegic patients found activity tuned to intended movements of all parts of the body, suggesting an integrative function^22^. Emerging work from Gordon et al. using a battery of MRI paradigms identifies three potential PCG regions breaking up otherwise somatotopic representation, which they propose coordinate whole-body action plans with specific connections to both striatal regions and the centromedian nucleus of the thalamus^23^. One of these identified regions may overlap with area 55b, and another, more superiorly positioned region, may overlie a secondary RMA region that we observed in five subjects (Extended Data Fig. 2). The interplay between these newly identified areas on the PCG convexity, the RMA area in the depths of the central sulcus and somatotopically delineated areas will be better understood by quantifying intracortical projections and efferents to motoneuronal cells in the spinal cord, which have been observed from both primary and association motor areas^24,25^. Future study to understand how the RMA has a wider role in motor circuitry might begin with more nuanced experimental paradigms that explore how this region interacts with primary motor regions and other motor association areas during speech production^26^, movement preparation^27^, motor imagery^28^, action observation^29^ and sensory feedback^30^.

## Methods

### Ethics statement

The study was conducted according to the guidelines of the Declaration of Helsinki and approved by the Institutional Review Board of the Mayo Clinic under IRB number 15-006530, which also authorizes sharing of the data. Each patient/representative voluntarily provided independent written informed consent to participate in this study as specifically described in the IRB review (with the consent form independently approved by the IRB).

### Participants

Thirteen patients (6 females, 11–20 years of age; Supplementary Table 1) participated in our study, each of whom underwent placement of 10–15 sEEG electrode leads for seizure network characterization in the treatment of drug-resistant partial epilepsy. No different experimental conditions were applied to the subjects and randomization was not possible. Data collection and analysis were not performed blind to the conditions of the experiments. Electrode locations were planned by the clinical epilepsy team based on typical semiology, scalp EEG studies and brain imaging. No plans were modified to accommodate research, nor were extra electrodes added. Thirteen of fifteen consecutive treated patients participated in our motor task. One excluded patient did not wish to participate in research (that is, did not consent) and the other excluded patient did not have appropriate pericentral electrodes. All experiments were performed at the Mayo Clinic in Rochester. Each patient or parental guardian provided informed consent as approved by the Institutional Review Board at Mayo Clinic (IRB, 15-006530). All T1 MRI sequences were defaced before uploading using an established technique^31^ to avoid potential identification. All subjects who consented to participate were recruited consecutively for 15 months. No statistical methods were used to predetermine sample sizes, but our sample sizes are similar to those reported in previous publications^3,15^. The subjects were not compensated for participation.

### Lead placement, electrode localization and referencing

The platinum depth electrode contacts (DIXI Medical) were 0.8 mm in diameter with 2 mm length circumferential contacts separated by 1.5 mm (Fig. 1 and Supplementary Fig. 1) (ref. ^32^). Each lead contained 10–18 electrode contacts. Surgical targeting and implantations were performed in the standard clinical fashion^32^. Intraoperatively, anchoring bolts were placed stereotactically in 2.3 mm holes in the skull, and leads were then advanced to target through the bolts. Once at target, leads were secured into the skull by a guide screw and cap (Fig. 1 and Supplementary Fig. 1).

Electrode anatomic localizations were determined by coregistration of postimplant CT scan to pre-implant MRI. Each preoperative T1 MRI was aligned to the anterior and posterior commissure stereotactic space (ACPC) using VistaSoft^33^, and the postimplant CT was registered to this ACPC-aligned T1 using mutual information in SPM12 (ref. ^34^). The electrode positions in the ACPC space were visualized on the T1 using a custom open-source MATLAB toolbox we developed (‘SEEGVIEW’)^35^.

All data were rereferenced in a bipolar fashion, producing channels that reflect mixed activity between voltage timeseries measured at two adjacent electrode contact sites. Plotted points for brain activity in this study represent an interpolated point between the two electrodes that make up each differential pair channel. Only adjacent differential pair channels were considered (that is, 1.5 mm from one another, on the same lead, and within the same lead segment for segmented leads; Supplementary Figs. 1 and 5). In each figure, channels were plotted using SEEGVIEW, which slices brain renderings and projects channels to the center of the closest slice^35^ in order to present analyses in an interpretable, clinically familiar manner. This projection approach imposes a longer projection distance if fewer/thicker slices are chosen for visualization. With fewer slices, all projected channels can be viewed more simply. Note that a channel reflecting activity in the gray matter at the depth of a sulcus may appear to be in white matter. Anatomic features (central sulcus, etc.) and designations of each channel were carried out by a neuroradiologist (T.J.K.).

### Motor task

Data were collected during a motor task involving (1) opening and closing of the hand, (2) side-to-side movement of the tongue with mouth closed and (3) alternating dorsi- and plantar flexion of the foot (contralateral to the hemisphere with pericentral sEEG electrode coverage). Subjects were visually cued to perform simple self-paced (~1 Hz) movements in response to images of a hand, tongue or foot, and to remain still during interleaved rest periods (blank black screen). Twenty cues (trials) of each movement type were shuffled in random order and move and rest cues were 3s in duration (Supplementary Fig. 2). This task was chosen based on prior work, which has produced clear results in recordings from the brain surface^36^. The BCI2000 software was used for stimulus presentation and data synchronization^37^, with stimuli presented on a 53 × 33 cm screen, 80–100 cm from the face (Supplementary Fig. 2). If subjects were not participating in the task, the experimental run would be stopped and rerun later.

### Electrical stimulation mapping

In subject 2, stimulation mapping was performed at the RMA site for clinical purposes. We found that bipolar stimulation up to 5 mA at the RMA site did not produce a sensory response and did not interrupt or elicit movement. Bipolar clinical stimulation at 2 mA produced contraction of the anterior tibialis at multiple foot somatotopic sites. Stimulation mapping was not performed in other subjects as it was not included in the research protocol although the IRB does allow the use of existing clinical data if it preserves the privacy of the patient (allowing us to review the stimulation data in subject 2).

### Electrophysiological recordings

Intracranial sEEG signals were initially recorded relative to a clinician-selected reference in the white matter away from tissue with likely seizure or motor involvement. Voltage timeseries were recorded with the 256-channel g.HiAmp amplifier (gTec). Recordings were sampled at 1,200 Hz, with an anti-aliasing filter, which dampened the signal by 3 dB at 225 Hz.

EMG was measured from the forearm flexors/extensors (hand), base of chin (tongue) and anterior tibialis (foot) during the motor task (Supplementary Fig. 3). All sEEG and EMG signals were measured in parallel, and delivered to both the clinical system and the research DC amplifier (g.HIAmp system, gTec). sEEG and EMG signals were synchronized with the visual stimuli using the BCI2000 software^37^.

### Signal processing and analysis

#### Trial-by-trial power spectral density calculations

All analyses were performed in MATLAB. Adjacent electrode contacts were first bipolar rereferenced to neighboring contacts on the same lead segment (Supplementary Fig. 5). To determine the precise timing of movement onset and offset in response to a visual cue, EMG-timing based analyses were chosen for behavioral analysis rather than the timing of the visual movement cue (Supplementary Fig. 6). EMG measuring tongue movement was lacking in subjects 5 and 6. In this case, we defined tongue movement periods by shifting all visual tongue cue onsets/offsets based on the subject-specific average delay between the onset/offset of cue and EMG activity for hand and foot movements. Within each movement trial, averaged power spectral densities (PSDs) were calculated from 1 Hz to 300 Hz every 1 Hz using Welch’s averaged periodogram method with 1 s Hann windows to attenuate edge effects^38^ and 0.5 s overlap. The averaged PSD for each movement or rest trial was normalized to the global mean across all trials. We normalized the PSDs in this way because brain signals of this type generally follow a 1/*f*, power law and shape^39^, so that lower frequency features dominate if un-normalized. From each of these normalized single trial PSDs, averaged power in a broadband high-frequency band (65–115 Hz) was calculated for subsequent analysis, as previously described^40^. This captures broadband activity above the known range of most oscillations and avoids ambient line noise at 60 Hz and 120 Hz. All steps of the basic spectral calculations are shown in Supplementary Fig. 4.

For each bipolar rereferenced channel, we calculated separate signed *r*^2^ cross-correlation values (*r*^2^) of the mean spectra from 65–115 Hz for each movement modality. Each channel’s *r*^2^ value was determined by comparing mean power spectra between movement trials (separately) and rest. To minimize the cross-effects of beta rebound, movement trials of each type were only compared with rest trials that followed that same movement type^41^:

$${r}_{{mr}}^{2}=\frac{{\left(\bar{m}-\bar{r}\right)}^{3}}{{{\rm{|}}\bar{m}-\bar{r}{\rm{|}}}{\sigma }^{2}_{m\cup r}}\frac{{N}_{m}{N}_{r}}{{N}_{m\cup r}^{2}}$$

where *m* denotes power samples from movement, *r* denotes samples from rest and the overline ($\bar{m}$ and $\bar{r}$) denotes sample mean. *m*$\cup$*r* represents combined movement and rest power sample distributions. *N*_*m*_ and *N*_*r*_ denote the total number of rest and movement samples and *N*_*m*_$\cup$_*r*_ = *N*_*m*_ + *N*_*r*_. Thus, *r*^2^ is the percentage of the variance in *m*$\cup$*r* that can be explained by a difference between the individual means in the subdistributions, $\bar{{\rm{m}}}$ and $\bar{{\rm{r}}}$. The sign indicates whether power is increasing or decreasing with movement. To calculate a *P* value for each channel and each movement type, we performed an unpaired two-sample *t*-test comparing broadband power for movement trials and the rest trials that immediately follow that movement type.

When viewing figures, consider that all channels were plotted at the interpolated position between the pairs measured, to reflect the change in the brain activity during movement versus rest. We chose a significance cutoff of one percent of the maximum *r*^2^ for all channels during a single modality, and insignificant channels were plotted with a white circle of fixed diameter.

#### Broadband timecourse analysis

sEEG broadband power timeseries was calculated by (1) band-passing the channel voltage with a third-order Butterworth filter in 10 Hz bands between 65 and 115 Hz, (2) applying the Hilbert transform and squaring each 10 Hz timeseries and (3) adding the 10 Hz timeseries together. The resulting signal was logged, *z*-scored, smoothed, exponentiated and centered at zero (that is, subtracting 1). EMG signal timeseries were band passed from 25 to 400 Hz (refs. ^42,43^) using a third-order Butterworth filter, notch filtered (60, 120, 180 Hz), enveloped and rectified. These were then logged, *z* scored, smoothed and exponentiated as in prior work^44^. All steps of the time series signal processing are shown in Supplementary Fig. 7.

Normalized broadband timeseries for somatotopic and RMA channels were averaged from 500 ms before to 4 s after movement onset (determined by manual annotation of the EMG). This normalization was done by summing the broadband timeseries of somatotopic channels during movement periods specific to the somatotopic tuning (for example, hand channels assessed during hand movement periods) and dividing by the total number of somatotopic channels: $\mathop{\sum }\nolimits_{k}^{N}{{BB}}_{k}(t)/N$, where *N* is the total number of hand channels and BB_k_(*t*) is the broadband timeseries for the *k*th channel. Data distribution was assumed to be normal, but this was not formally tested, and are shown in Fig. 3h and Supplementary Figs. 15 and 18–20.

### Somatotopic tuning and shared representation in each channel

Note that the rare negative *r*^2^ values (that is, broadband decreases with movement) were set to zero before calculation of both somatotopic tuning and shared representation.

#### Somatotopic delineation

Somatotopic delineation for a specific movement was calculated for each channel in the following manner. The individual *r*^2^ values for each movement type were multiplied by ${e}^{{\rm{i}}{{\pi }}/6}$ (hand), ${e}^{i{{\pi }}5/6}$ (tongue), ${e}^{i{{\pi }}3/2}$ (foot) and added together. The magnitude of the resulting complex number defines the strength of somatotopic selectivity, and the phase angle of the complex number points to the movement (or pair of movements) that the channel is somatotopically specific for. This is illustrated in Figs. 1e and 2a. Note that only channels exceeding 50% of the within-subject maximum magnitude for somatotopic selectivity are included in the plot of Fig. 2a.

#### Shared representation

We calculated overlap of movement representation for each channel as the geometric mean of significant *r*^2^ values (*P* values < 0.05 by unpaired *t*-test) of all three movement types: ${{\mathrm{overlap}}}=\sqrt[3]{{r}_{H}^{2}\cdot {r}_{T}^{2}\cdot {r}_{F}^{2}\,}\,$. Note that only channels exceeding 50% of the within-subject maximum overlap are included in the plot of Fig. 2b.

#### Estimation of timing between brain activity and movement

To estimate the relative latencies between brain activity and movement (Fig. 3h), we calculated cross correlations between channels of sEEG and EMG signals. These correlations were calculated by taking the dot product of an sEEG channel’s broadband timecourse and the rectified EMG timecourse measuring hand, tongue and foot movement. Correlations were measured after introducing time delays ranging from −2 s to 2 s, in 1 sample (0.83 ms) intervals, obtaining a profile of correlation as a function of latency between the two signals (that is a ‘sliding window’ to calculate correlation, Fig. 3g).

### Group Level Analyses

#### Transforming ACPC electrode coordinates to Montreal Neurological Institute (MNI152) space

Electrode coordinates in standard MNI152 space were obtained by first calculating the nonlinear unified segmentation-based normalization of the T1 scan in SPM12 (ref. ^45^). The ACPC to MNI152 transformation was then applied to the electrode positions. MNI coordinates on the right hemisphere were all reflected to the left hemisphere for visualizations at the group level. For group analyses in MNI152 space, a Rolandic, pericentral, volume slab was delineated to select a subset of recording sites, as illustrated in Supplementary Fig. 8. This delineation was done by selecting lines anterior, posterior and inferior to the precentral and postcentral gyri. Distances from the cortical convexity were obtained by first generating a convex hull of the MNI152 brain left hemisphere (as described previously^46^), and then identifying the closest point on the hull to each channel site (illustrated in Supplementary Fig. 9).

#### *K*-means clustering

*K*-means clustering^47^ was performed to identify natural groups in the data independent of our hypotheses. First, a 3D feature space was constructed with signed *r*^2^ values of broadband high-frequency change for foot, hand and tongue on the *x*, *y* and *z* axes, respectively. Each channel was plotted as a point in this space, and subjects 1–11 were included together. Subjects 12–13 were excluded because of poor data quality. Subject 2 was included twice (once for left-sided movements and once for right-sided movements).

*K*-means clustering was then performed on this feature space for cluster sizes of 1–20 to determine the appropriate cluster number, and a total error versus cluster number tradeoff was measured. As illustrated in Supplementary Fig. 10, if the simple tradeoff (penalty) function $N* \sum _{k}{D}_{k}^{2}$ is used (where $N$ is number of clusters and ${D}_{k}^{2}$ is the Euclidean distance of channel $k$ to the center of the cluster it is assigned to), a global minimum cannot be determined because high cluster numbers are overly favored. However, appeal to the elbow method^48^ suggests that five would be the appropriate number of clusters. If ${N}^{2}* \sum _{k}{D}_{k}^{2}$ is instead used as a penalty function, low cluster number is favored. A middle-ground penalty function ${N}^{1/2}* \sum _{k}{D}_{k}^{2}$ exhibits a good tradeoff between number of clusters and error, and clustering was repeated 1,000 times over a range of 1–20 clusters, taking the minimum as the optimal number of clusters. A histogram of number of times each number of clusters was selected shows that five is the best number of clusters. The clustering with five clusters that produced the minimum error ${D}_{k}^{2}$ across the 1,000 repetitions was selected as the optimal clustering for use in this study.

The optimal clustering, shown in Fig. 2 and Supplementary Fig. 10, reveals that these clusters correspond to channels that do not become active for any movement type and are selective for hand, foot or tongue, and those with shared representation across all three channels (RMA).

Clustering was performed in an identical manner for an 8–32 Hz lower frequency (beta) rhythm range, as shown in Supplementary Fig. 11. As might be expected in similar work from work in ECoG^36^, this produced only two clusters.

#### Decoding algorithm using linear discriminant analysis

To examine how well RMA channels distinguish between trials of different movements and rest, and compare this to the performance of somatotopic sites, a decoding analysis was performed (Supplementary Fig. 14). First, *P* values (by unpaired *t*-test) were calculated at each channel for each movement type by comparing movement trials to the rest periods that followed that movement (as was done for *r*^2^ values). RMA channels were defined as those with *P* < 0.05 for each movement type independently. Somatotopic channel sites were defined as those with *P* < 0.01666 (that is *P* < 0.05 Bonferroni corrected for three movement types) for only one movement type. Sites significant for two movement types (adjacent/overlapping representation) and sites with no significant values were not included in classification.

The classification was performed using linear discriminant analysis with threefold cross-validation, separately for RMA and somatotopic channels. All movement and rest trials were used in classifier training, and accuracies were reported separately for classification of all trials and just movement trials. Sub-classification by training with just movement trials was not possible because the limited number of trials in that case leaves the calculated covariance matrix underdetermined during training.

### Reporting summary

Further information on research design is available in the Nature Portfolio Reporting Summary linked to this article.

## Online content

Any methods, additional references, Nature Portfolio reporting summaries, source data, extended data, supplementary information, acknowledgements, peer review information; details of author contributions and competing interests; and statements of data and code availability are available at 10.1038/s41593-023-01346-z.

### Supplementary information

Supplementary Information Supplementary Table 1 and Supplementary Figs. 1–33.

Reporting Summary

## Figures and Tables

**Extended Data Fig. 4:**
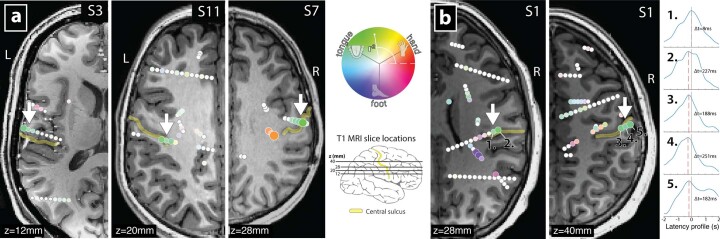
Characteristics of tongue-selective somatotopic sites. **(a)**. The majority of tongue-selective somatotopic sites lie anterior or within the central sulcus, as seen for Subjects 3 (left), 11 (middle), & 7 (right). **(b)**. In Subject 1, we see that tongue-selective somatotopic sites may be present in both the anterior (numbered 1-3) and posterior (numbered 4-5) banks of the central sulcus, and these sites all precede EMG activity (when evaluated using latency profiles). Interestingly, if latency calculations for somatotopically-selective and RMA sites are limited only to the precentral gyrus (Supplementary Fig. 15), latencies are comparable to the full distribution across all sites (Fig. 3h).

## Data Availability

All data recorded necessary to interpret, verify and extend the research are publicly available at: https://osf.io/p5n2k. No data were excluded from this set and all data are anonymized. A README document is found in the link above, which includes a detailed description of all variables included in the data files.

## Code availability

All code to perform analyses and reproduce the illustrations are publicly available at: https://osf.io/p5n2k.
