# Author Correction: A motor association area in the depths of the central sulcus

**DOI:** 10.1038/s41593-024-01693-5

**Published:** 2024-06-04

**Authors:** Michael A. Jensen, Harvey Huang, Gabriela Ojeda Valencia, Bryan T. Klassen, Max A. van den Boom, Timothy J. Kaufmann, Gerwin Schalk, Peter Brunner, Gregory A. Worrell, Dora Hermes, Kai J. Miller

**Affiliations:** 1 Medical Scientist Training Program, Mayo Clinic, Rochester, MN USA; 2 Neurosurgery, Mayo Clinic, Rochester, MN USA; 3 Department of Physiology and Biomedical Engineering, Mayo Clinic, Rochester, MN USA; 4 Neurology, Mayo Clinic, Rochester, MN USA; 5 Radiology, Mayo Clinic, Rochester, MN USA; 6 Chen Frontier Lab for Applied Neurotechnology, Tianqiao and Chrissy Chen Institute, Shanghai, China; 7 Neurosurgery, Fudan University/Huashan Hospital, Shanghai, China; 8 Neurosurgery, Washington University School of Medicine, St Louis, MO USA

**Keywords:** Motor cortex, Sensorimotor processing, Motor control

Correction to: *Nature Neuroscience* 10.1038/s41593-023-01346-z, published online 18 May 2023

In the version of the article initially published, the equation in the “Trial-by-trial power spectral density calculations” section of Methods was incorrect; the sigma in the denominator was preceded by a superscript 3 and should have been followed by a superscript 2. The correct equation is shown below:

$${r}_{mr}^{2}=\frac{{(\overline{m}-\overline{r})}^{3}}{|\overline{m}-\overline{r}|{\sigma }_{m\cup r}^{2}}\frac{{N}_{m}{N}_{r}}{{N}_{m\cup r}^{2}}$$

The equation has been corrected in the HTML and PDF versions of the article.

