# Supplementary material for: A motor association area in the depths of the central sulcus
Source: Nat Neurosci. 2023 May 18;26(7):1165–9. doi: 10.1038/s41593-023-01346-z (PMC10322697; doi:10.1038/s41593-023-01346-z)
Supplement: Supplementary file 1 — Supplementary Table 1 and Supplementary Figs. 1–33. [file 41593_2023_1346_MOESM1_ESM.pdf]

nature neuroscience

Brief Communication https://doi.org/10.1038/s41593-023-01346-z

A motor association area in the depths of the central sulcus

In the format provided by the authors and unedited

SUPPLEMENTARY MATERIAL

For the manuscript **"A motor association area in the depths of the central sulcus"**

By Michael A. Jensen, Harvey Huang, Gabriela Ojeda Valencia, Bryan T. Klassen, Max A. van den Boom, Timothy J. Kaufmann, Gerwin Schalk, Peter Brunner, Gregory A. Worrell, Dora Hermes, Kai J. Miller

Supplementary Tables & Figures:

Methodological Illustrations: Illustrations Corresponding to Methods Section

Table 1: Table of Subject Information- to see demographic and clinical data for each subject (Page S2)

Figure 1: Configuration of sEEG- to visualize volumetric nature of sEEG leads (Page S3)

Figure 2: Task Design- to understand timing of visual cues during our motor task (Page S4)

Figure 3: EMG recording sites- photographs of EMG sticker placement sites (Page S5)

Figure 4: Basic Spectral Calculations- to see processing workflow up to spectral analysis (Page S6)

Figure 5: Plotting Interpolated Pairs- to see how the data from a bipolar-referenced channel are plotted (Page S7)

Figure 6: EMG vs. Visual-cue Segmentation- to see the segmentation method's impact on motor maps (Page S8)

Figure 7: Time Series Signals- to see processing workflow for EMG and sEEG time series (Page S9)

Figure 8: Selection of Peri-Central Recording Sites- delineation of the peri-central volume slab (Page S10)

Figure 9: Depth Measurement- measuring a recording site's distance from the surface of a convex hull (Page S11)

Figure 10: Optimizing Cluster No. for K-means Algorithm- how cluster number was determined naively (Page S12)

Additional Results: Discretionary Data and Analyses Not Shown in Main Text

Figure 11: Beta Band K-means- clustering with r^2 values using power from 8-32Hz (Page S13)

Figure 12: Motor Maps using Beta Band (Subject 4)- coronal slices of channels significantly active during hand tongue and foot movement based on low frequency oscillations r^2 values (Page S14)

Figure 13: Primary RMA Site in Subjects 12 & 13 (Low-fidelity data) as in Extended Data Fig. 1 (Page S15)

Figure 14: Decoding Algorithm- comparison of accuracy between somatotopic and RMA sites (Page S16)

Figure 15: Histograms of latency between somatotopic hand/foot/tongue sites from the precentral gyrus (PCG) and their paired EMG traces (Page S17)

Illustration of findings in individual subjects:

Broadband Timecourse of RMA and Somatotopic Channels

Figures 16 – 18: Replication of Main Text Figure 3 (A-E) plus latencies for Subjects 1, 2, and 4 (Pages S18-S120)

Peri-Central Motor Maps with somatotopic delineation and shared representation

Figures 19– 33: Peri-central Broadband-based Motor Mapping for Subjects 1-13 (Pages S21-S35)

Subject ID	Age	Sex	# Leads/Side	EMG Recorded	Somatotopic Coverage in PCG	Seizure Foci and Lesional Notes	Handedness
S1	16	F	14/R	H,T,F	H T F	**Lesion:** No lesion<br>**SOZ:** Right precentral postcentral gyri and paracentral lobule	L
S2[%]	16	M	15/B	H,T,F	H F	**Lesion:** Focal Cortical Dysplasia (FCD) in frontal lobe<br>**SOZ:** Frontal lobe near FCD	L
S3	16	F	14/L	H,T*,F	H T F	**Lesion:** No lesions<br>**SOZ**: Operculum insula and post central gyrus	R
S4	15	F	10/L	H,T,F	H T	**Lesion:** Left mesial temporal lobe sclerosis<br>**SOZ**: L mesial temporal lobe	R
S5	17	F	13/R	H ,F**	H	**Lesion:** Right temporal low-grade glioma<br>**SOZ**: Right mesial temporal (amygdala, hippocampal body and post. hippocampus), right middle temporal gyrus and right insula	R
S6	13	M	13/R	H,F[◇]	H F	**Lesion:** No lesions<br>**SOZ:** Right temporal	R
S7	20	M	12/R	H,T,F	H T	**Lesion:** Right posterior temporal lobe (prior resection)<br>**SOZ:** Post Par-Occ-Temp junction (previous resection), Anterolateral Temporal Lobe<br>Mesial Temporal Lobe	R
S8	14	F	15/L	H,T,F	N/A	**Lesion:** Left inf frontal cortical thickening and blurring of grey-white junction at base of gyrus rectus/orbital gyrus<br>**SOZ:** Left anterior insula/ inferior frontal junction	R
S9	18	M	14/R	H,T,F	H F	**Lesion:** Right mesial temporal lobe sclerosis<br>Anterior temporal lobectomy<br>**SOZ:** Right posterior temporal lobe and insula	L[♪]
S10	18	F	15/L	H,T,F	N/A	**Lesion:** Left occipital and posteromedial temporal: atrophy, cortical/subcortical calcification, and leptomeningeal enhancement.<br>**SOZ (15 seizures):** Left temporo-parieto-occipital (9), diffuse (5), post. Temporal (1)	R
S11	11	M	12/L	H,T,F	H T F	**Lesion:** No lesions<br>**SOZ:** Paracentral Lobule	R
S12[†%]	12	M	15/R	H,T,F	H	**Lesion:** Posterior paramedian<br>**SOZ multifocal:** Inf occipital, lingual gyrus, hippocampal	R
S13[†]	13	M	11/B	H,T,F	H	**Lesion:** No lesions<br>**SOZ:** Right frontal region seizures are generalized. Lennox Gastaut	R

Supplementary Table 1. Subject Information: Age, Sex, Number of Leads/Laterality, EMG modalities with useful signal, somatotopic tuning represented in the precentral gyrus (PCG), seizure foci location, and handedness. [*]Noise contaminated EMG signal in initial trials; [**]Noise contaminated EMG signal in all trials; [◇]Patient refused to have chin EMG stickers placed; [†]S12 and S13 had low quality data; [♪]R language dominant; [%]Left/Right sided movement within same subject (R indicates right sided movement). Note: the cluster not shown is the null cluster, containing the remaining channels for each patient.

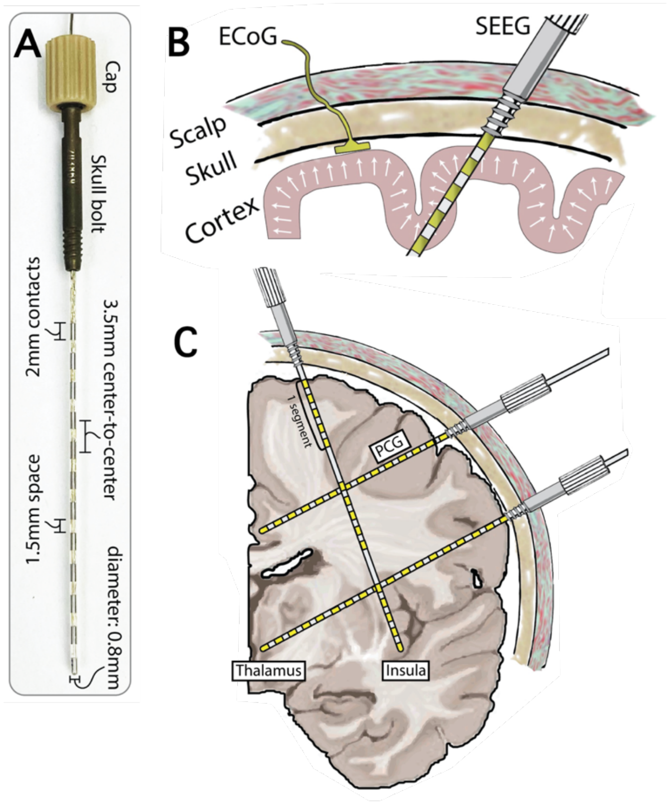

Supplementary Figure 1. Configuration of sEEG electrodes. A. Labeled specifications of sEEG electrodes implanted into subjects. **B.** Schematic illustrating the sulcal coverage provided by sEEG and the limitation of ECoG electrodes to the brain's contour. **C.** Hemispheric coronal slice showing segmented and non-segmented sEEG leads with the ability to record from the entire brain volume.

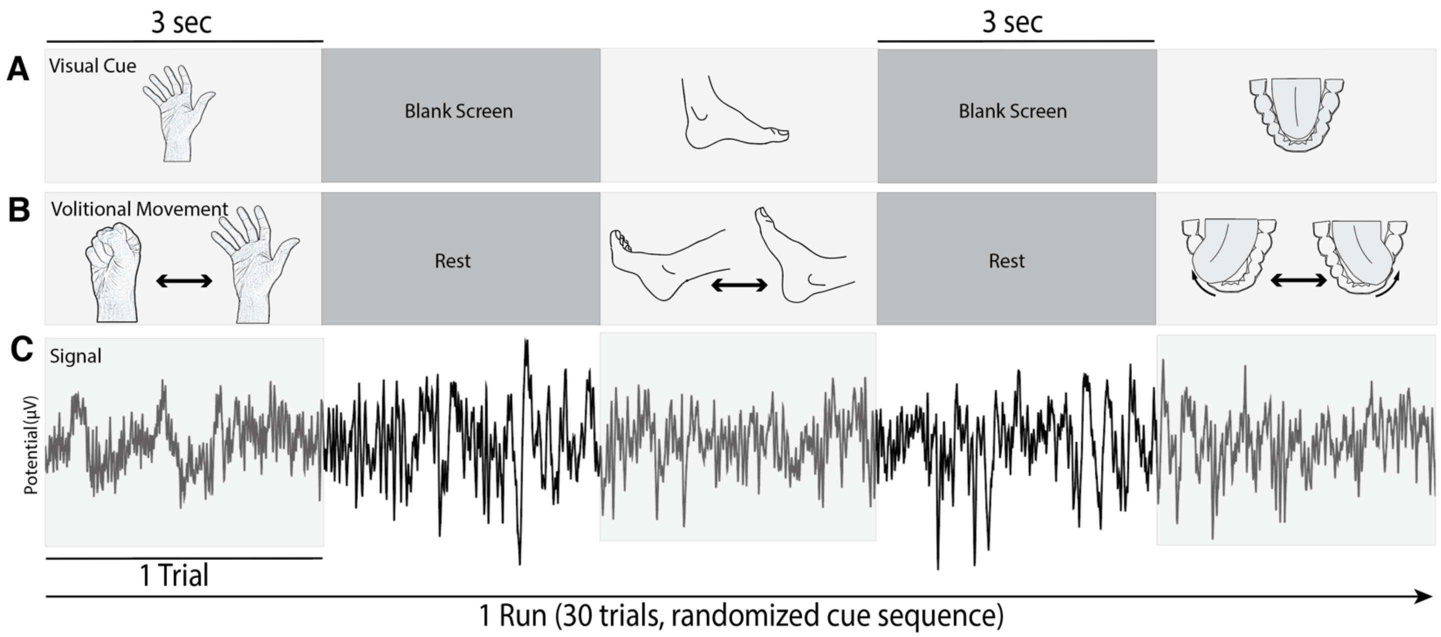

Supplementary Figure 2. Motor Task Design. A. Subjects were presented with a 3 second visual cue on a screen 75-100 cm away displaying either a hand, foot, or tongue with a blank screen interleaved. **B.** Subjects were instructed to open and close their hand, dorsi and plantar flex their foot, and move their tongue laterally with mouth closed upon visualization of cue of hand, foot, tongue respectively. Subjects were instructed to remain still when the screen was blank. **C.** sEEG signal was recorded across all trials and the entire set of trials (hand, foot, or tongue movement periods) in a single run. There were 30 trials per run.

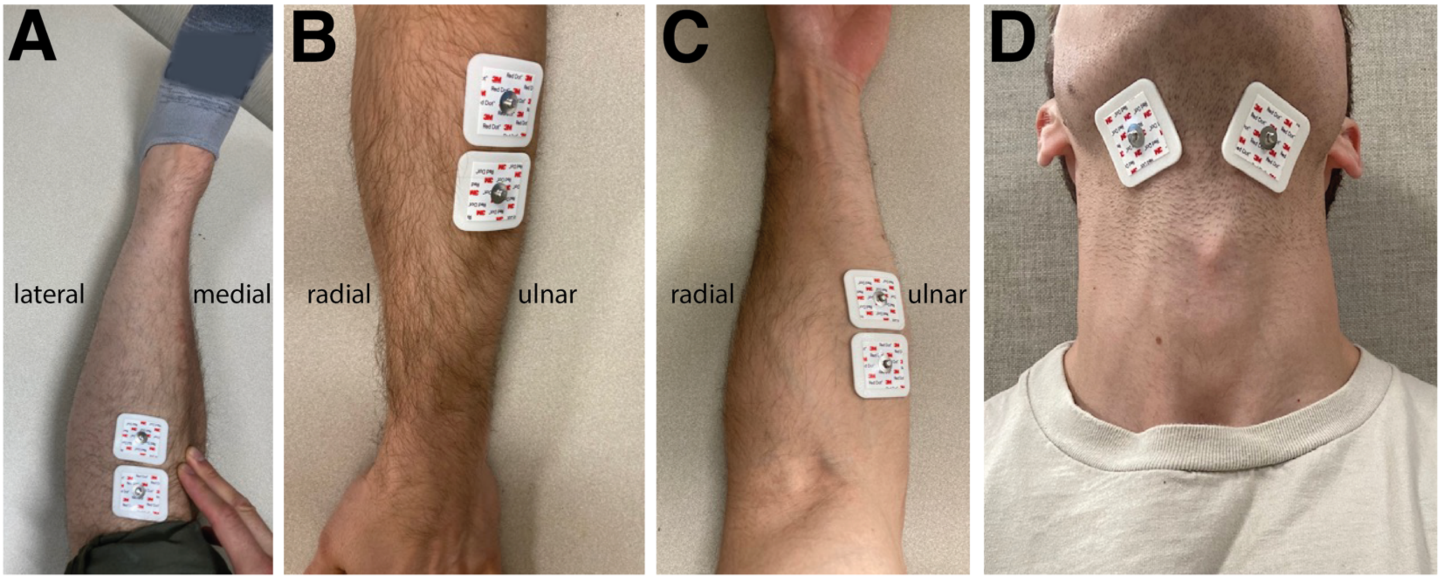

Supplementary Figure 3. EMG Sticker Placement. A. Foot (i.e. anterior tibialis) **B.** Hand (i.e. extensor carpi ulnaris, extensor digitorum) **C.** Forearm Flexors (i.e. flexor carpi radialis). **D.** Tongue (i.e. suprahyoid muscles).

Subject 6

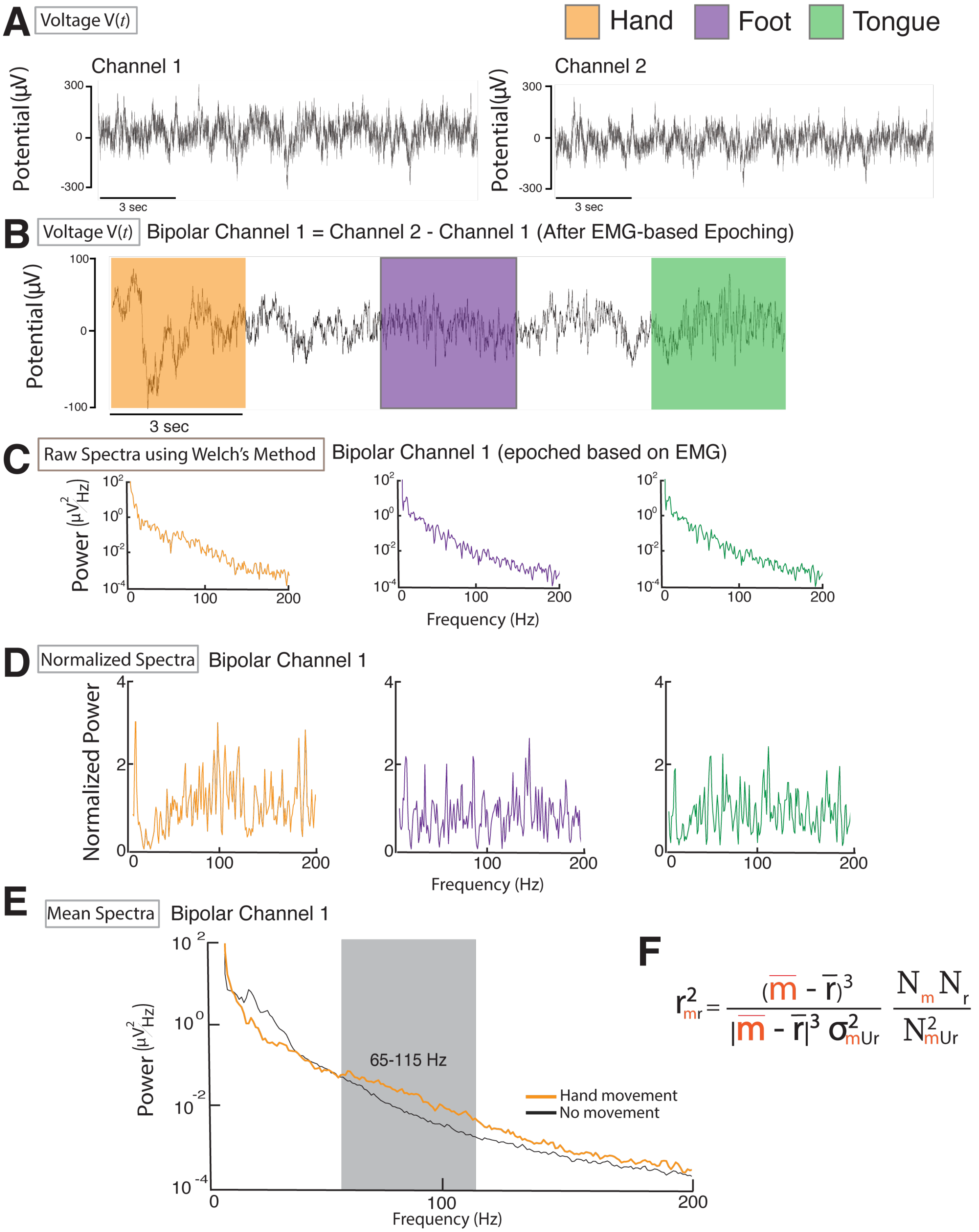

Supplementary Figure 4. Basic Spectral Calculations. A. Adjacent channels within the same segment of the same lead were re-referenced in a bipolar fashion. **B.** Voltage time series were segmented using EMG-defined movement periods. **C.** The power spectral density of each trial was calculated. **D.** Spectra were normalized to the mean spectra and logged. **E.** Average power spectra for all trials of the same movement type (e.g. mean of hand trials) were used to compare 65-115Hz (HFB) power between movement and rest. **F.** r^2 values were calculated using the mean HFB power during trials of a single movement type and the rest periods immediately following.

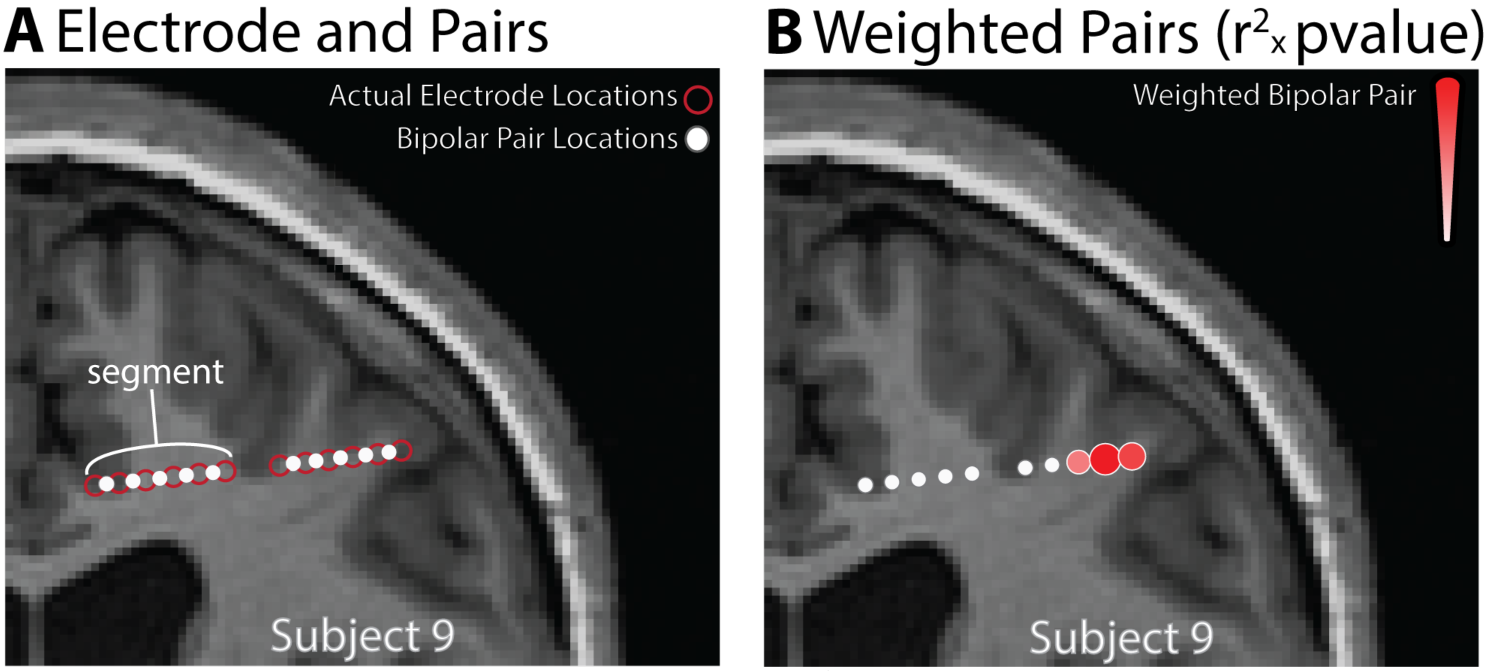

Supplementary Figure 5. Electrode plots reflect bipolar channel pairs and r^2 values. A. Plotted circles represent the interpolated points between the two electrode channels making up a differential pair. **B.** The sign and magnitude of the r^2 correlation coefficient determines the size and color hue of each electrode pair plotted.

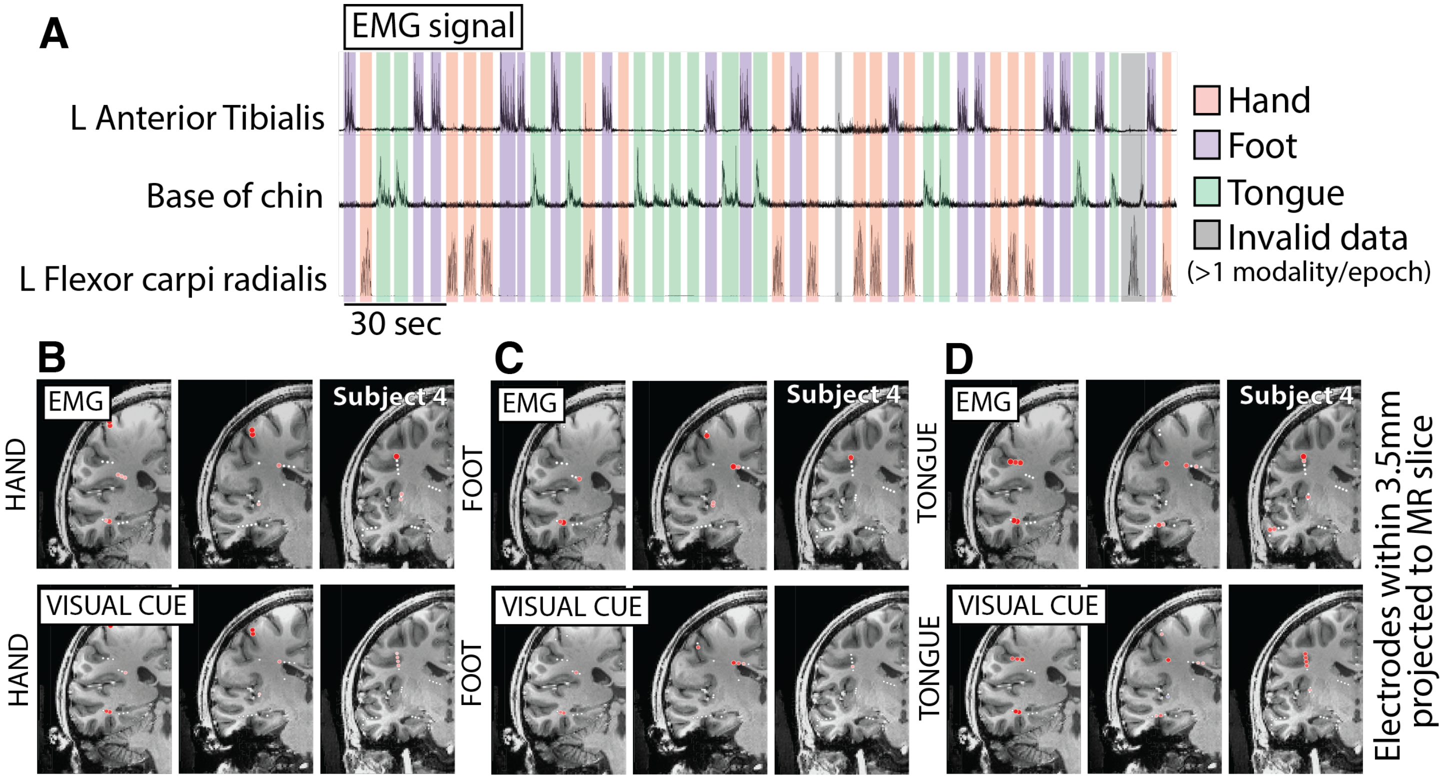

Supplementary Figure 6. EMG vs. cue-based segmentation. A. EMG signals measured between pairs of channels placed as seen in Supplementary Figure 3. Trials specific to hand, foot, or tongue are shaded in orange, purple, and green respectively with gray demonstrating epochs with simultaneous movement of multiple modalities. The absence of background shading indicates resting. **B-D.** Comparison of results between EMG and stimulus-based data segmentation for hand, foot, and tongue movement in Subject 4. Note that maps differ between the two segmentation methods.

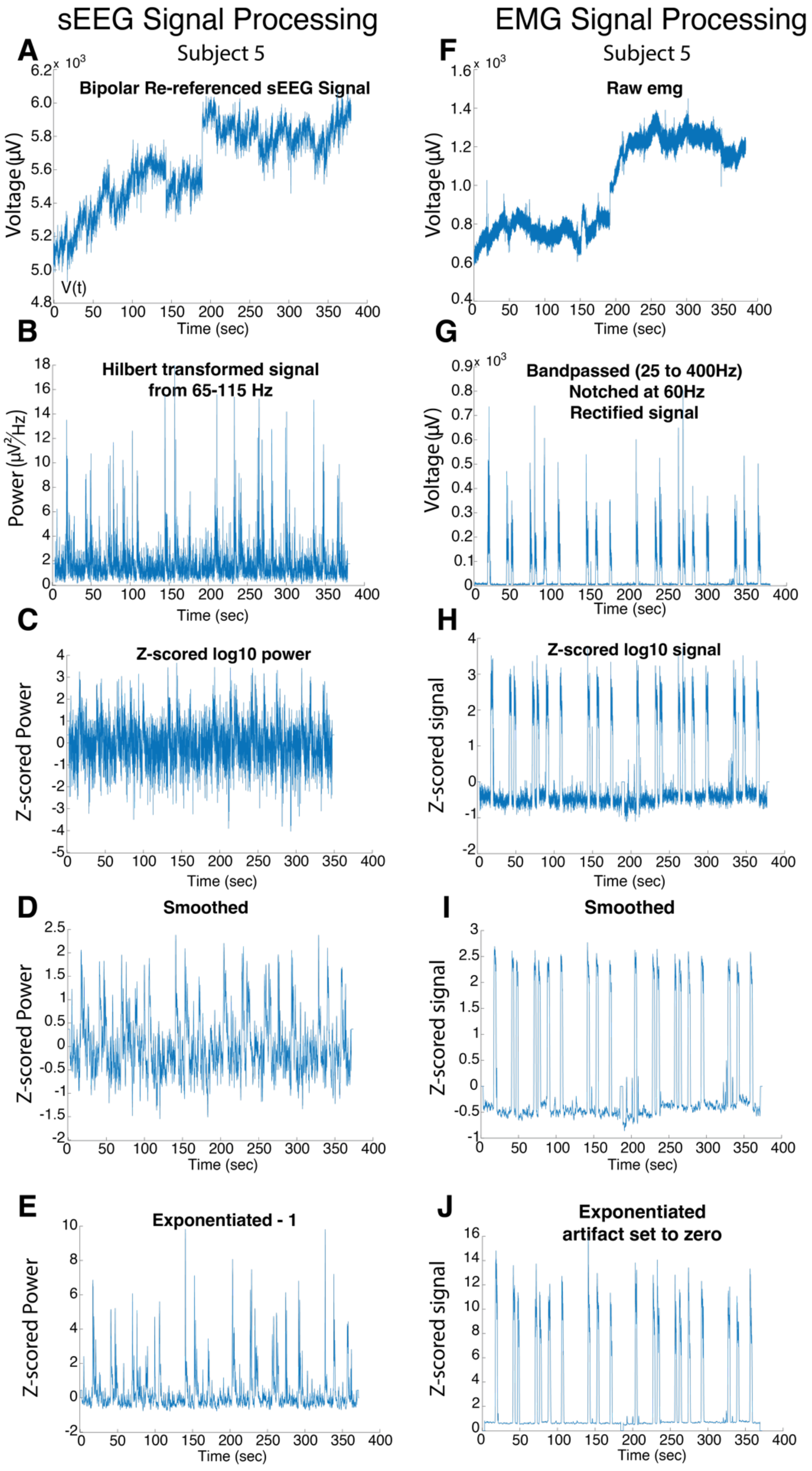

Supplementary Figure 7. Time Series Signal Processing. Subject 5 A. The sEEG signal were bipolar re-reference **B.** The sEEG signal was band-passed using a 3rd order Butterworth filter in 10Hz bands (from 65-115Hz), Hilbert transformed {Hilbert(V(t)) = V(t) + iH(t) = $re^{i\Omega}$}, squared, and the 10Hz timeseries were added together. The signal was then **(C)** logged and z scored, **(D)** smoothed with a moving average window of 500ms, and then **(E)** exponentiated and centered around zero by subtracting 1. **F.** The EMG was recorded in a bipolar fashion, then **(G)** band-passed from 25 to 400Hz, **(H)** logged and z scored, **(I)** smoothed with a moving average window of 500ms, and then **(J)** exponentiated. Inter-run artifact was set to zero.

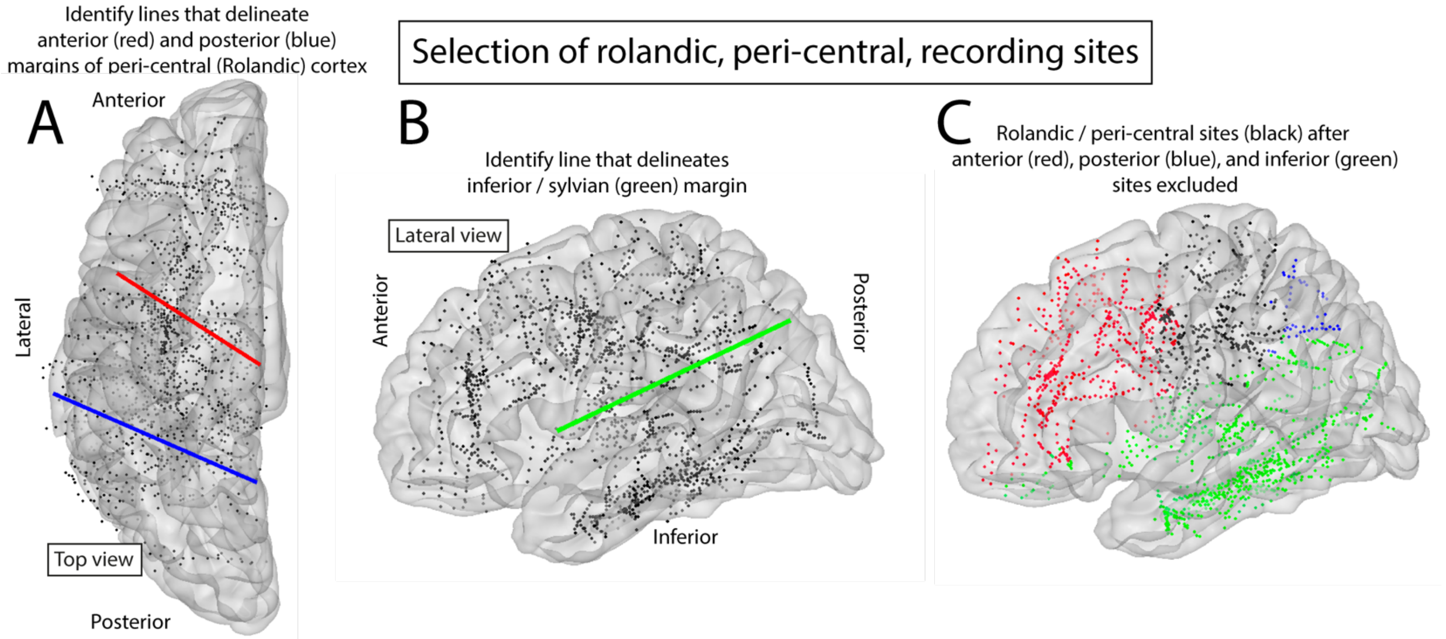

Supplementary Figure 8. Seletion of the peri-central (Rolandic) recording sites in the MNI152 brain left hemisphere. A. Identification of lines that delineate the anterior (red) and posterior (blue) boundaries of the peri-central volume slab. **B.** Identification of the line that delineates the inferior boundary of the peri-central volume slab. **C.** All channels plotted on a common MNI brain rendering (anterolateral view) with channels in the peri-central sensorimotor cortex colored black. Channels outside of the sensorimotor cortex are color coded based on their respective exclusion boundaries. Keep in mind this figures uses red, green, and blue to represent channels outside the peri-central volume and not neural acitvity.

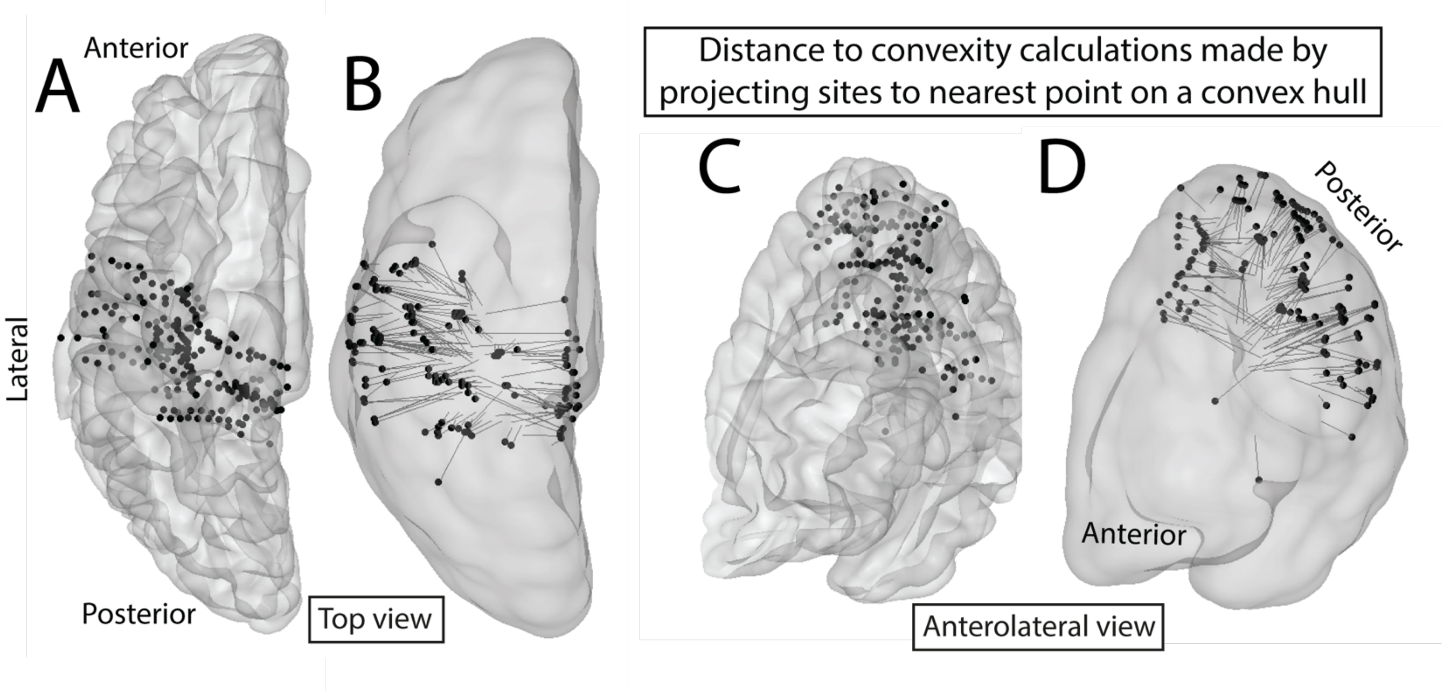

Supplementary Figure 9. Measuring recording sites' distance from the cortical convexity. A. Peri-central recording sites from all subjects plotted in the left hemisphere of the common MNI152 brain rendering shown from above. **B.** The recording sites in A are projected to the nearest vertex of a convex hull of the MNI152 left hemisphere brain rendering. The projection distance to the hull convexity serves as the depth measurement of each recording site. **C.** As in A, but from an anterolateral view. **D.** As in B, from an anterolateral view.

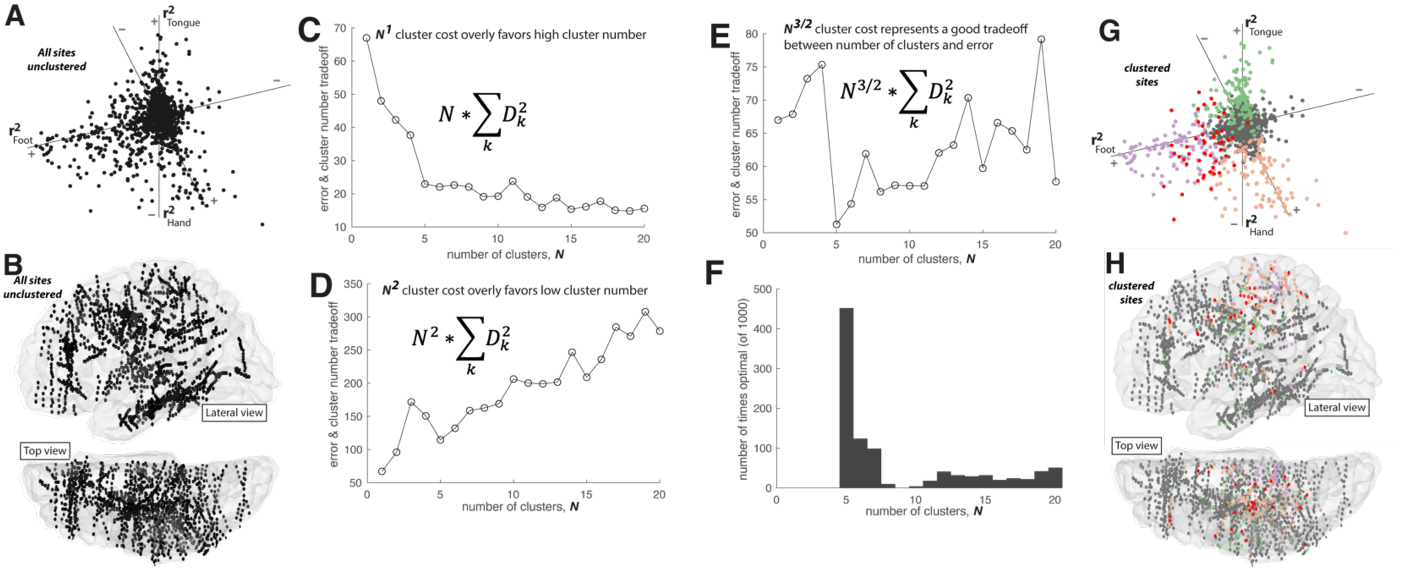

Supplementary Figure 10. Naively Identifying K-means Cluster Number. A. We generated a 3-dimensional feature space with signed r^2 values of broadband high frequency change for foot, hand, and tongue on the x-, y-, and z-axes, respectively. **B.** Visualization of all channels from all subjects on a common MNI brain rendering left hemisphere. **C.** Clustering was performed the feature space in A for cluster sizes of 1 to 20 to determine the appropriate cluster number, and the total error vs. cluster number tradeoff was measured. Using a simple tradeoff (penalty) function $N * \sum_k D_k^2$ a global minimum cannot be determined since high cluster numbers are overly favored. However, the Elbow Method[39] suggests that 5 would be the appropriate number of clusters. **D.** Using the tradeoff function $N^2 * \sum_k D_k^2$ a low cluster number is favored. **E.** A middle-ground penalty function $N^{3/2} * \sum_k D_k^2$ exhibits a good tradeoff between number of clusters and error. **F.** Clustering using the function from E 1000 times for a range of 1 to 20 clusters, we see that 5 clusters minimizes the tradeoff error in nearly half of the iterations. Based on this, and the plateau at 5 clusters in C[39] we chose to set K to 5. **G.** As in A, color coded by cluster. **H.** As in B, channels color coded by cluster.

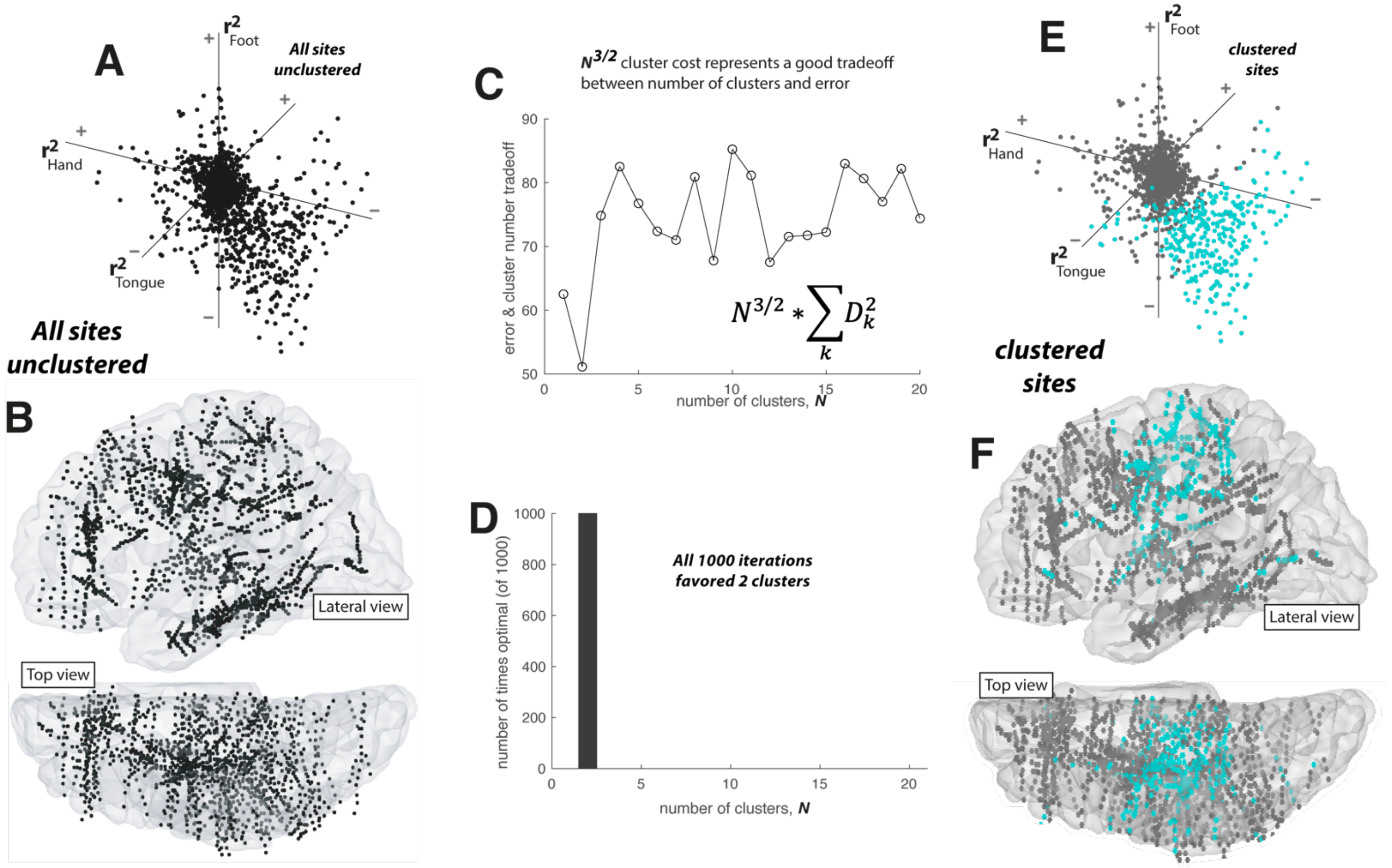

Supplementary Figure 11. Naively identifying channel ensembles using K-means Clustering using a low frequency band (8-32Hz, LFB). A. We generated a 3-dimensional feature space with signed r^2 values LFB power change for foot, hand, and tongue on the x-, y-, and z-axes, respectively **B.** Visualization of all channels from all subjects on a common MNI brain rendering left hemisphere. **C.** The middle-ground penalty function $N^{3/2} * \sum_k D_k^2$ used in Supplementary Figure 10 was applied to the LFB r^2 values and a minimum was identified. **D.** Across one-thousand iterations, error was minimized at 2 clusters. **E.** As in A, color coded by cluster. **F.** As in B, channels color coded by cluster. We see that these clusters generally separate sensorimotor cortex from the rest of the brain but do not distinguish between movement types.

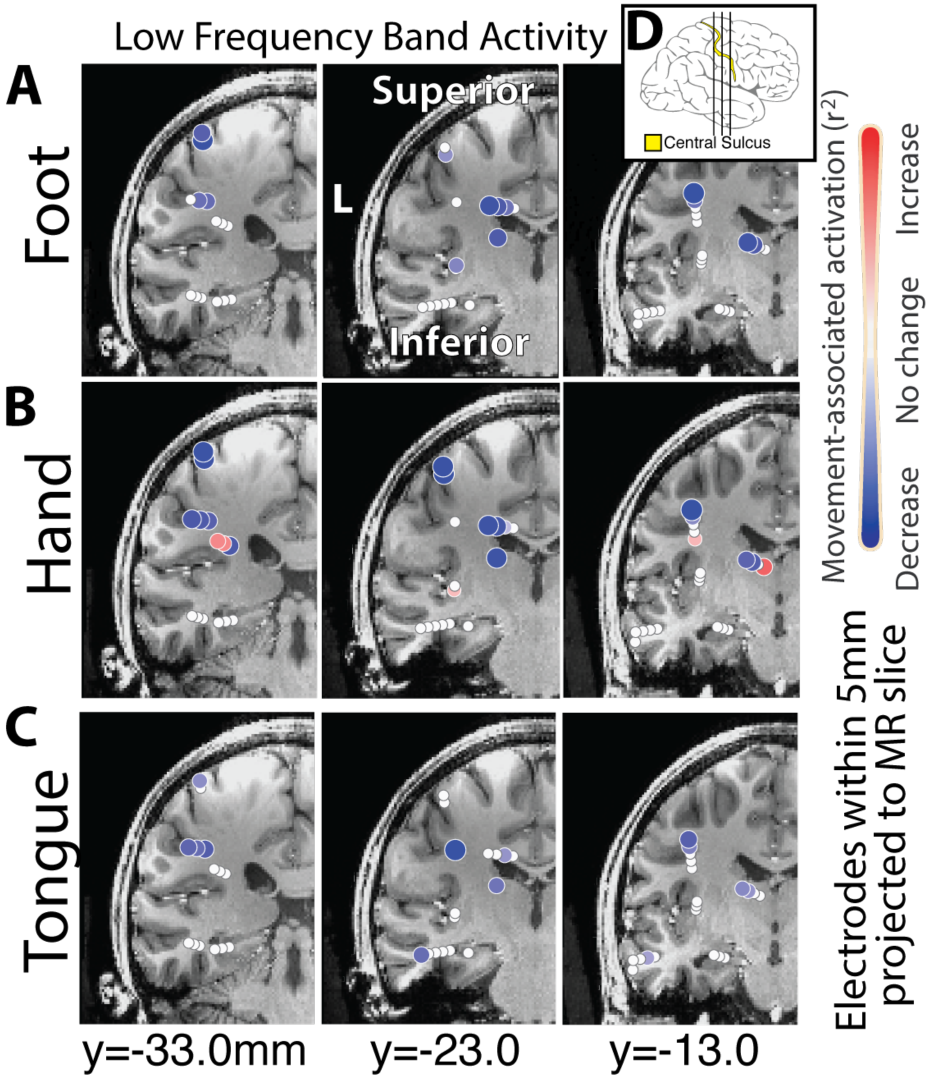

Supplementary Figure 12. **Using power changes from 8-32 Hz to map motor representation (Subject 4).** Axial T1 MRI cross sections through central sulcus with differential electrode pair channels (circles) brain areas with significant decreases from in power from 8-32Hz during movement (r^2 values) during foot (**A**), hand (**B**), and tongue (**C**) movement. A, B, C have extensive overlap, eliminating the ability to distinguish somatotopic representation. **D.** Coronal slices throughout the central sulcus and precentral gyrus shown with black lines.

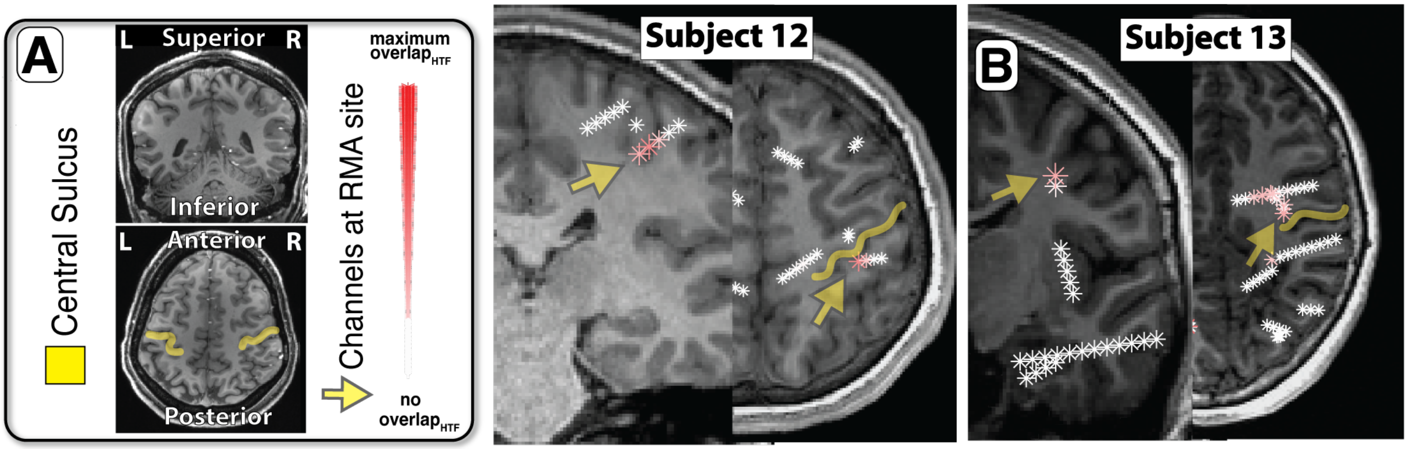

Supplementary Figure 13. Rolandic motor association area within the central sulcus: subjects with low-fidelity data. Coronal and axial slices of overlapping electrode pairs between hand, tongue, and foot movement for Subjects 10 & 11. Axial and coronal views demonstrate localization of overlapping pairs within the central sulcus. Due to low quality of task compliance (e.g. fidgeting, myoclonic bursts) there were very few quality trials. The results are similar, but the fidelity of these data are low as the trial numbers are few.

A

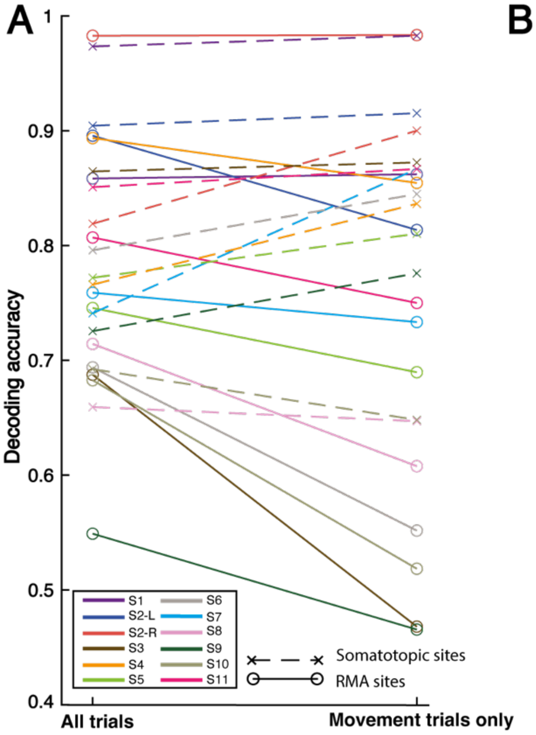

B

Subject	Somatotopic sites accuracy		RMA sites accuracy	
	All trials	Movement trials only	All trials	Movement trials only
1	0.973	0.982	0.858	0.862
2-L	0.904	0.915	0.895	0.813
2-R	0.818	0.900	0.982	0.983
3	0.864	0.872	0.68	0.468
4	0.765	0.836	0.893	0.854
5	0.771	0.810	0.745	0.689
6	0.795	0.844	0.693	0.551
7	0.741	0.866	0.758	0.733
8	0.659	0.647	0.714	0.607
9	0.725	0.775	0.549	0.465
10	0.692	0.648	0.682	0.518
11	0.850	0.866	0.807	0.750

all trials: Rest, Hand, Tongue, and Foot

movement trials only: only Hand, Tongue, and Foot

Supplementary Figure 14. Decoding trials (rest, hand, tongue, and foot) from RMA & somatotopic channels using linear discriminant analysis with 3-fold cross-validation A. Line plots of decoding accuracy using data from RMA or somatotopic channels within each subject. **B.** RMA sites have higher decoding accuracy for all (rest, hand, tongue, and foot) trials compared to only movement (hand, tongue, and foot) trials ($p = 0.0016$). Somatotopic sites have higher decoding accuracy for only movement (hand, tongue, and foot) trials compared to all (rest, hand, tongue, and foot) trials ($p = 0.0272$). Somatotopic sites have higher decoding accuracy than RMA sites for only movement (hand, tongue, and foot) trials ($p = 0.0051$). Somatotopic sites have higher decoding accuracies for all (rest, hand, tongue, and foot) trials than RMA sites, but the difference is not statistically significant ($p = 0.4470$).

The results of this classification (Supplementary Fig. 14) show that somatotopic sites have higher decoding accuracy than RMA sites for only movement (hand, tongue, and foot) trials. Sub-classification shows that somatotopic channels distinguish between different types of movement better than they do all trials together (i.e. when rest trials are included). RMA channels show the opposite – they are better at classifying rest from movement than they are at distinguishing between movements. Presumably, this is because when decoding using data from somatotopic sites (e.g. hand site) all non-hand trials (foot, tongue, or rest) would be indistinguishable, while data from RMA channels provides useful information for each trial type. P-values are shown in the figure caption, calculated using a paired t-test.

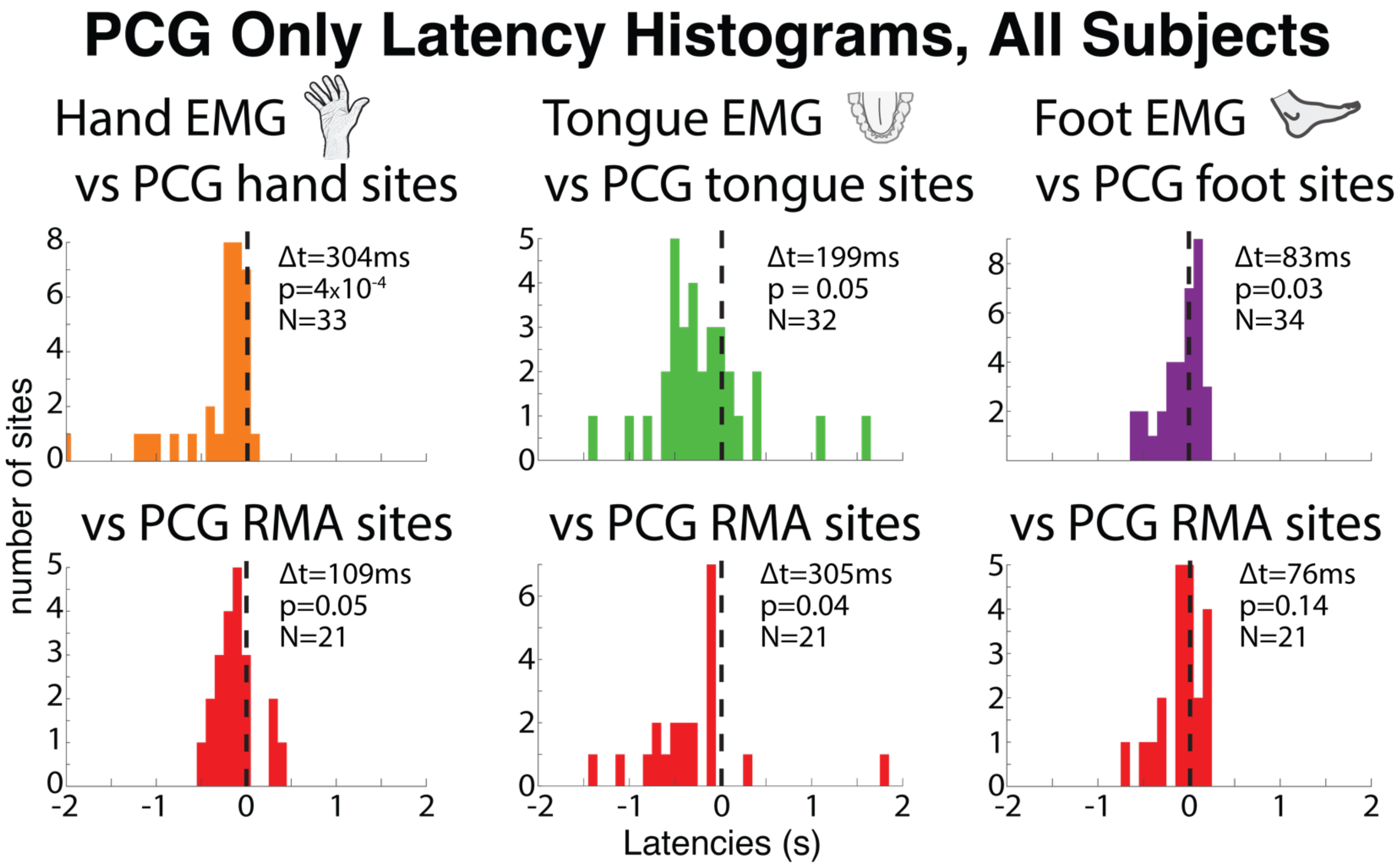

Supplementary Figure 15: Histograms of latency between somatotopic hand/foot/tongue sites from the precentral gyrus (PCG) and their paired EMG traces, as well as latencies of PCG RMA sites to each EMG trace (as in Fig 3H, but constrained to the PCG). For these clustered sites, localization to the PCG was performed by projection into the MNI atlas brain and was then visually segmented by a neuroradiologist (TJK) who was blinded to cluster label.

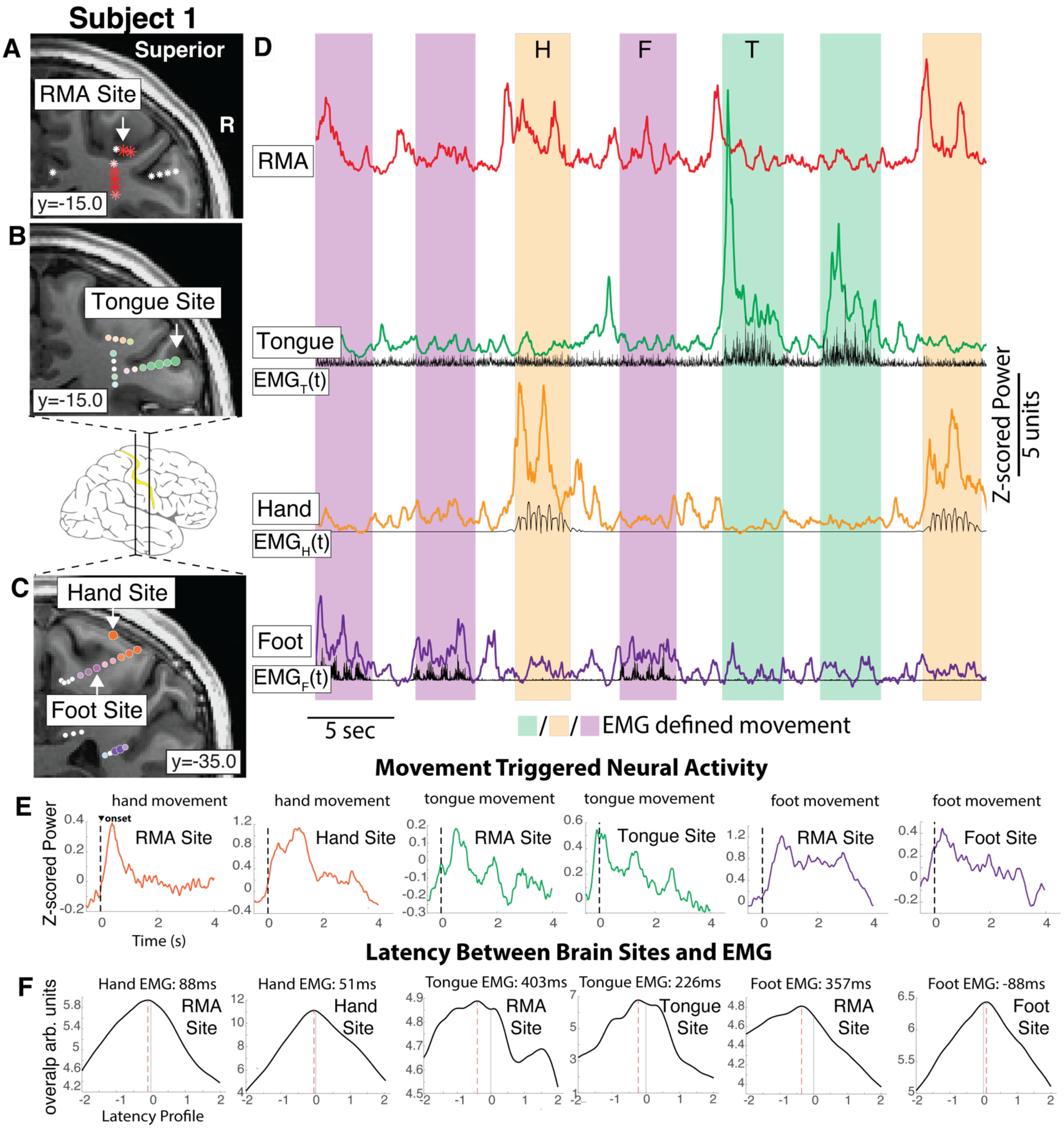

Supplementary Figure 16. Temporal dynamics of neural activity in the precentral gyrus, subject 1. A. Coronal T1 MRI cross section through PCG in the left hemisphere, with plotted shared activity revealing the RMA area. **B.** Left hemisphere, with somatotopic tongue representation. **C.** Left hemisphere, with somatotopic foot and hand representation. **D.** Timecourse of broadband activity (65-115Hz) reflecting local neural activity for sites with the highest overlap (RMA) and somatotopic (hand, tongue, foot) indices. Background shading indicates EMG-defined movement periods of the hand (orange), tongue (green) and foot (purple). **E.** Brain activity averaged to onset of foot and hand EMG and tongue stimulus onset. As the signal from each channel is normalized to itself, the units do not aid in comparison of magnitude. **F.** Latency profiles between brain the RMA, tongue, hand, and foot site broadband power and hand, tongue (after zeroing low-fidelity periods), and foot EMG.

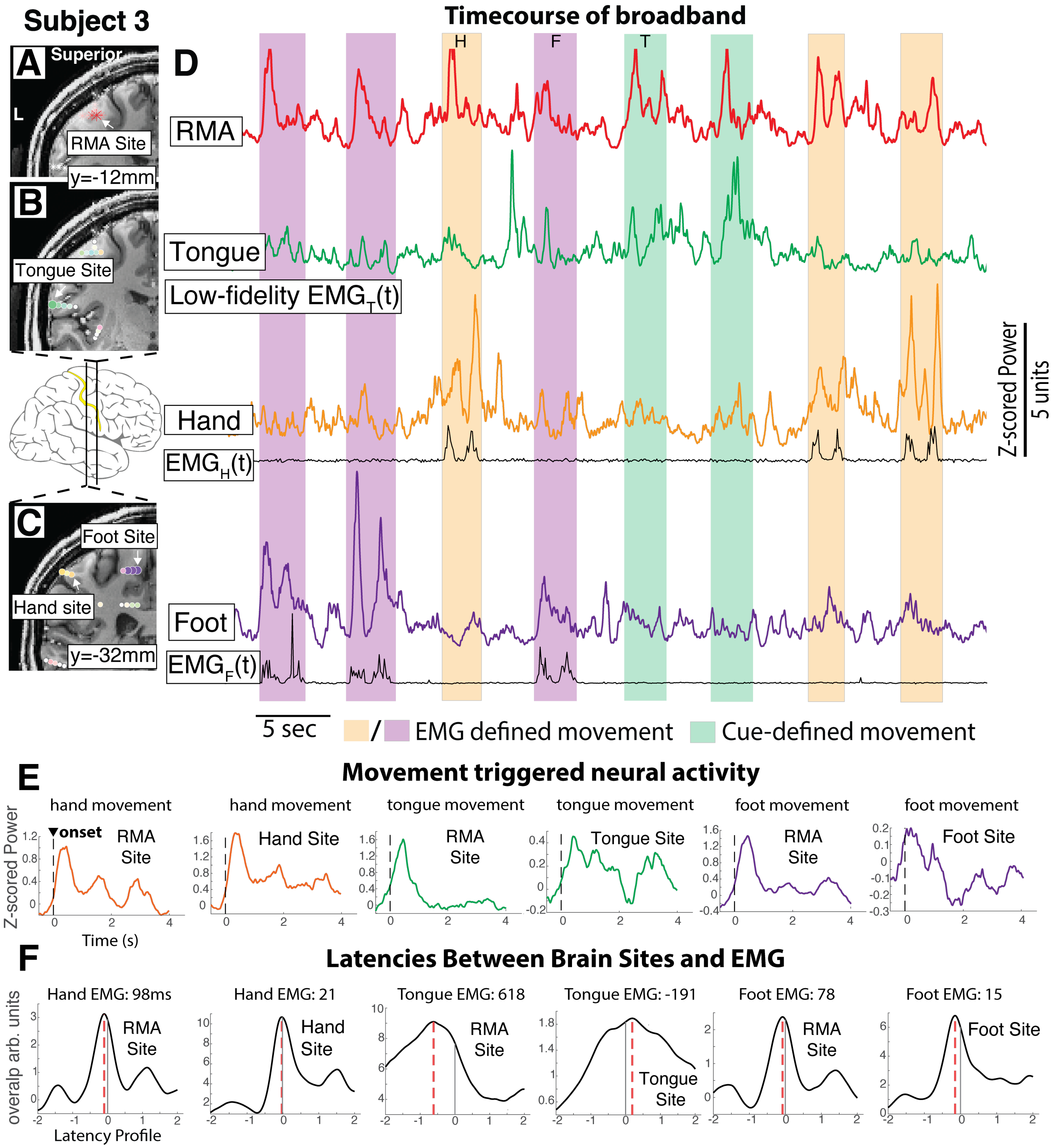

Supplementary Figure 17. Temporal dynamics of neural activity in the precentral gyrus, subject 3. A. Coronal T1 MRI cross section through PCG in the left hemisphere, with plotted shared activity revealing the RMA area. **B.** Left hemisphere, with plotted somatotopic tongue representation. **C.** Left hemisphere, with plotted somatotopic foot and hand representation. **D.** Timecourse of broadband activity (65-115Hz) reflecting local neural activity for sites with the highest overlap (RMA) and somatotopic (hand, tongue, foot) indices. Background shading indicates EMG-defined movement periods of the hand (orange) and foot (purple). Note: High fidelity tongue EMG were lacking for these trials, so green shading indicates stimulus cue timing, **E.** Brain activity averaged to onset of foot and hand EMG and tongue stimulus onset. As the signal from each channel is normalized to itself, the units do not aid in comparison of magnitude. **F.** Latency profiles between brain the RMA, tongue, hand, and foot site broadband power and hand, tongue (after zeroing low-fidelity periods), and foot EMG.

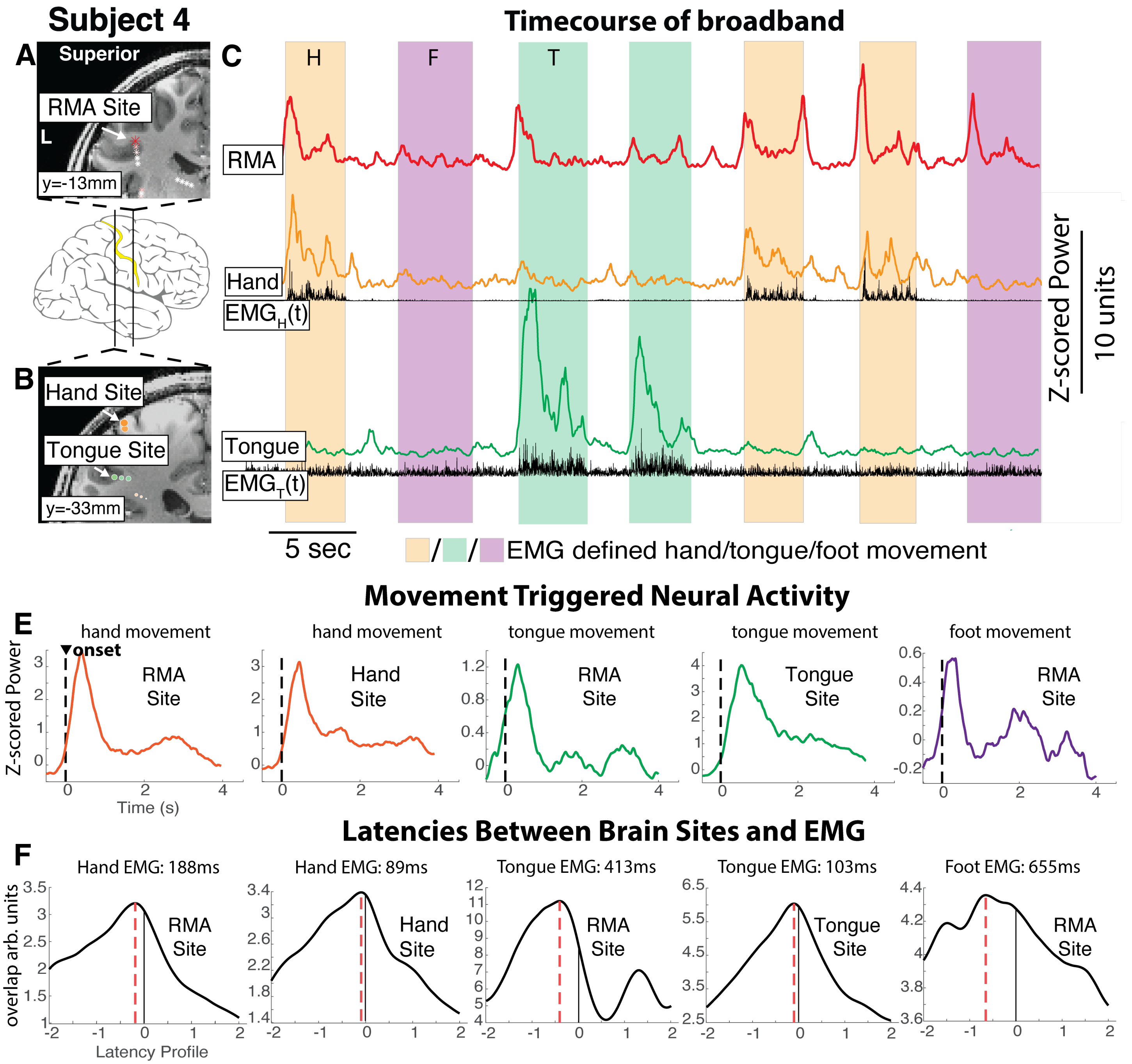

Supplementary Figure 18. Temporal dynamics of neural activity in the precentral gyrus, subject 4. A. Coronal T1 MRI cross section through the precentral gyrus (PCG) and central sulcus in the left hemisphere, with plotted shared activity revealing the RMA site. **B.** Left hemisphere, with plotted somatotopic hand and tongue representation. **D.** Timecourse of broadband activity (65-115Hz) reflecting local neural activity for sites with the highest overlap (RMA) and somatotopic (hand & tongue) indices. Background shading indicates EMG-defined movement periods of the hand (orange), tongue (green), and foot (purple). **E.** Brain activity averaged to onset of movement for the sites with highest overlap (RMA site) and somatotopic (hand and tongue sites) indices. As the signal from each channel is normalized to itself, the units do not aid in comparison of magnitude. Note: There is no somatotopic foot representation in this subject **F.** Latency profiles between brain areas (RMA site, tongue site, hand site) and EMG (hand, tongue, and foot).

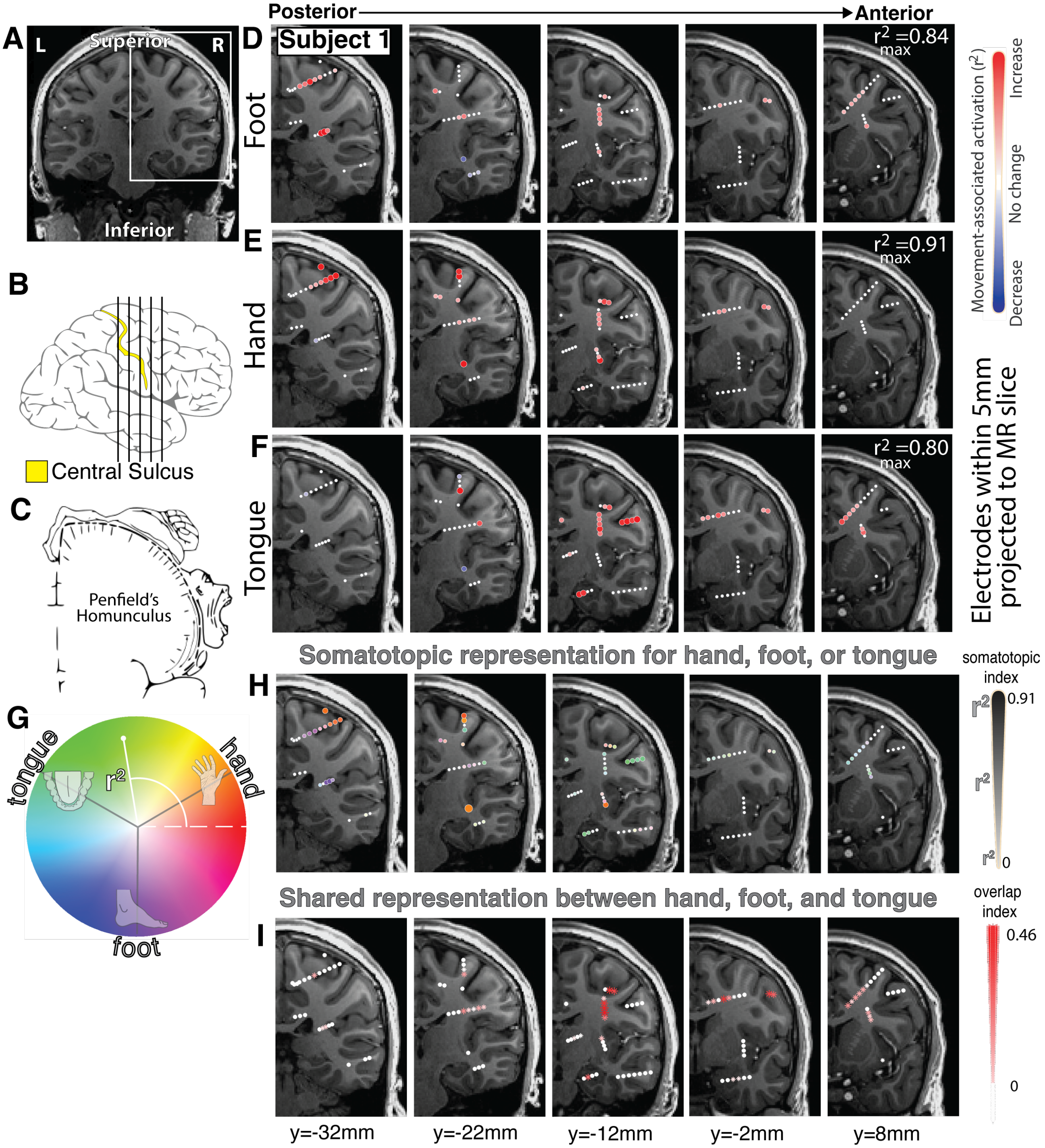

Supplementary Figure 19. Volumetric electrophysiological changes during simple body movements. A. Coronal T1 MRI, with inset box assessed throughout figure. **B.** Coronal slices throughout central sulcus / precentral gyrus shown with black vertical lines. **C.** The classic Penfield motor homunculus from awake stimulation shown as reference[1]. **D-F.** Maps of sEEG power spectral change in the 65-115Hz range during foot, hand, and tongue movement, respectively. Maximum scaling of the color bar is noted in the top right of each row. **G.** Circular colormap showing the color scheme used to indicate somatotopic tuning (H). Color reflects the presence of somatotopic tuning while diameter and intensity indicate the magnitude. Note that a channel that is equally active (even if highly so) during all 3 movement types will be plotted small and white, even if strongly tuned to each movement. **H.** Movement somatotopic maps using scale from (G). **I.** Maps of shared activity in movement (geometric mean of hand, tongue, and foot r^2 values). Insignificant channels are plotted in white in all panels.

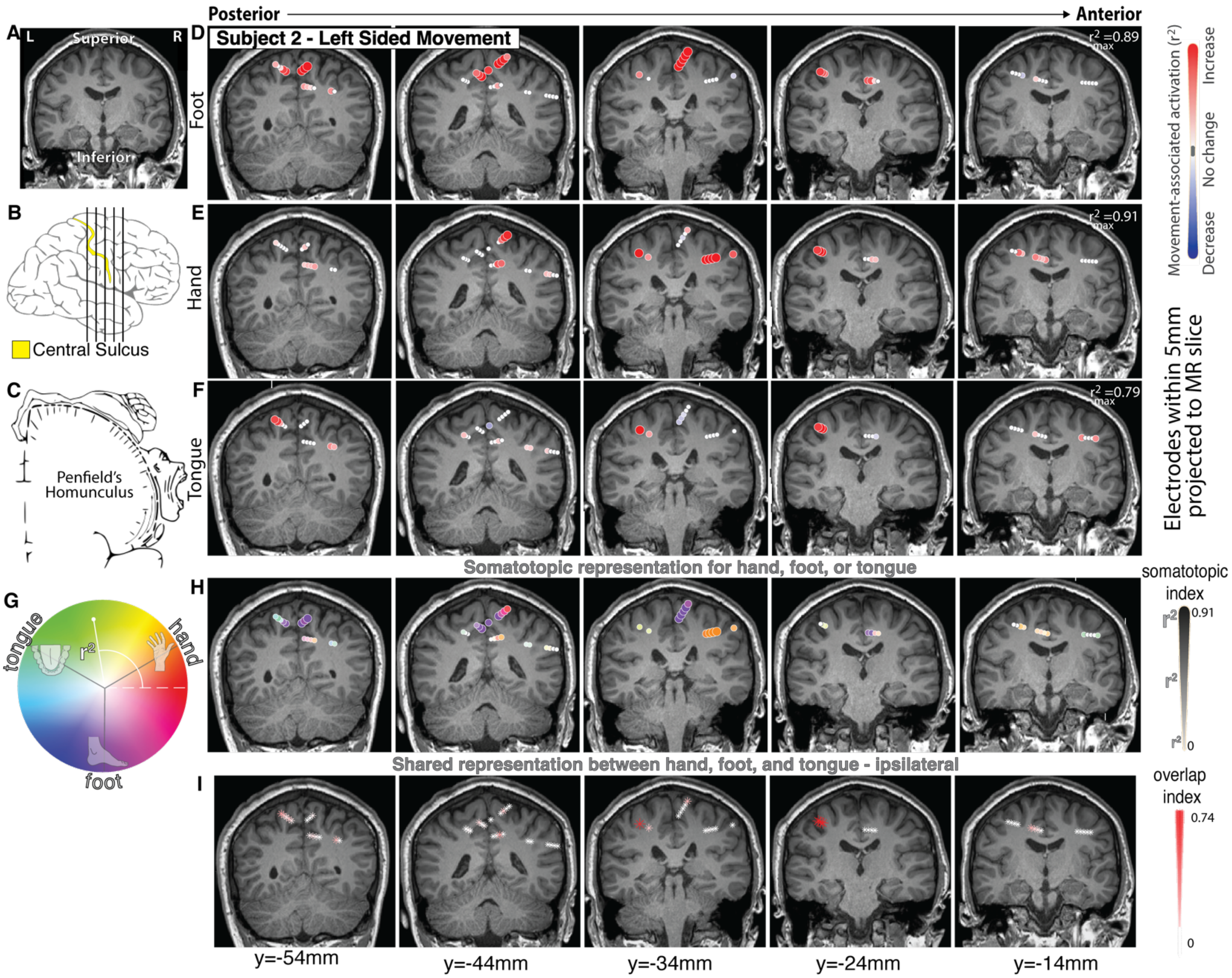

Supplementary Figure 20. Volumetric electrophysiological changes during simple body movements. A. Coronal T1 MRI, with inset box assessed throughout figure. **B.** Coronal slices throughout central sulcus / precentral gyrus shown with black vertical lines. **C.** The classic Penfield motor homunculus from awake stimulation shown as reference[1]. **D-F.** Maps of sEEG power spectral change in the 65-115Hz range during foot, hand, and tongue movement, respectively. Maximum scaling of the color bar is noted in the top right of each row. **G.** Circular colormap showing the color scheme used to indicate somatotopic tuning (H). Color reflects the presence of somatotopic tuning while diameter and intensity indicate the magnitude. Note that a channel that is equally active (even if highly so) during all 3 movement types will be plotted small and white, even if strongly tuned to each movement. **H.** Movement somatotopic maps using scale from (G). **I.** Maps of shared activity in movement (geometric mean of hand, tongue, and foot r^2 values). Insignificant channels are plotted in white in all panels.

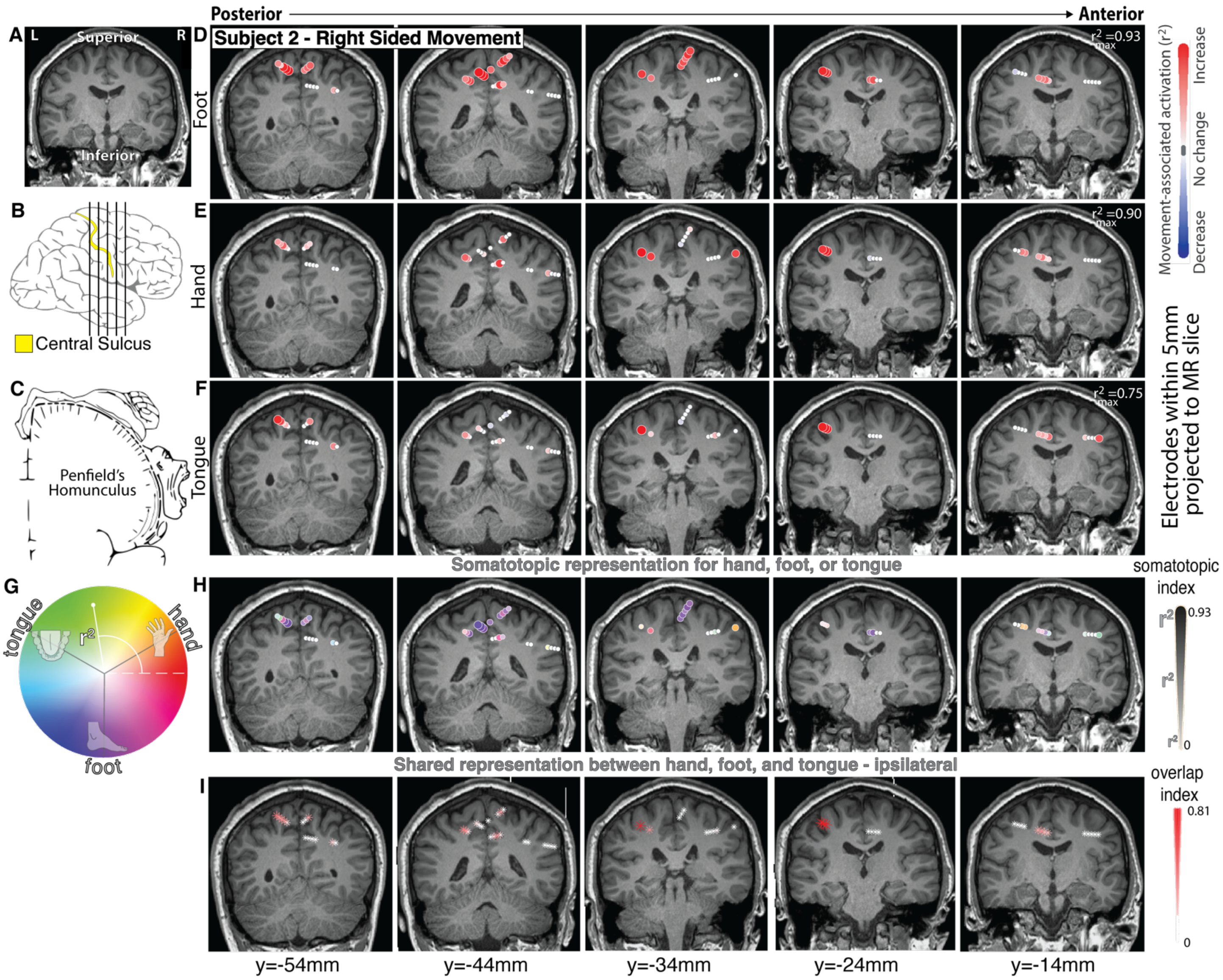

Supplementary Figure 21. Volumetric electrophysiological changes during simple body movements. A. Coronal T1 MRI, with inset box assessed throughout figure. **B.** Coronal slices throughout central sulcus / precentral gyrus shown with black vertical lines. **C.** The classic Penfield motor homunculus from awake stimulation shown as reference[1]. **D-F.** Maps of sEEG power spectral change in the 65-115Hz range during foot, hand, and tongue movement, respectively. Maximum scaling of the color bar is noted in the top right of each row. **G.** Circular colormap showing the color scheme used to indicate somatotopic tuning (H). Color reflects the presence of somatotopic tuning while diameter and intensity indicate the magnitude. Note that a channel that is equally active (even if highly so) during all 3 movement types will be plotted small and white, even if strongly tuned to each movement. **H.** Movement somatotopic maps using scale from (G). **I.** Maps of shared activity in movement (geometric mean of hand, tongue, and foot r^2 values). Insignificant channels are plotted in white in all panels.

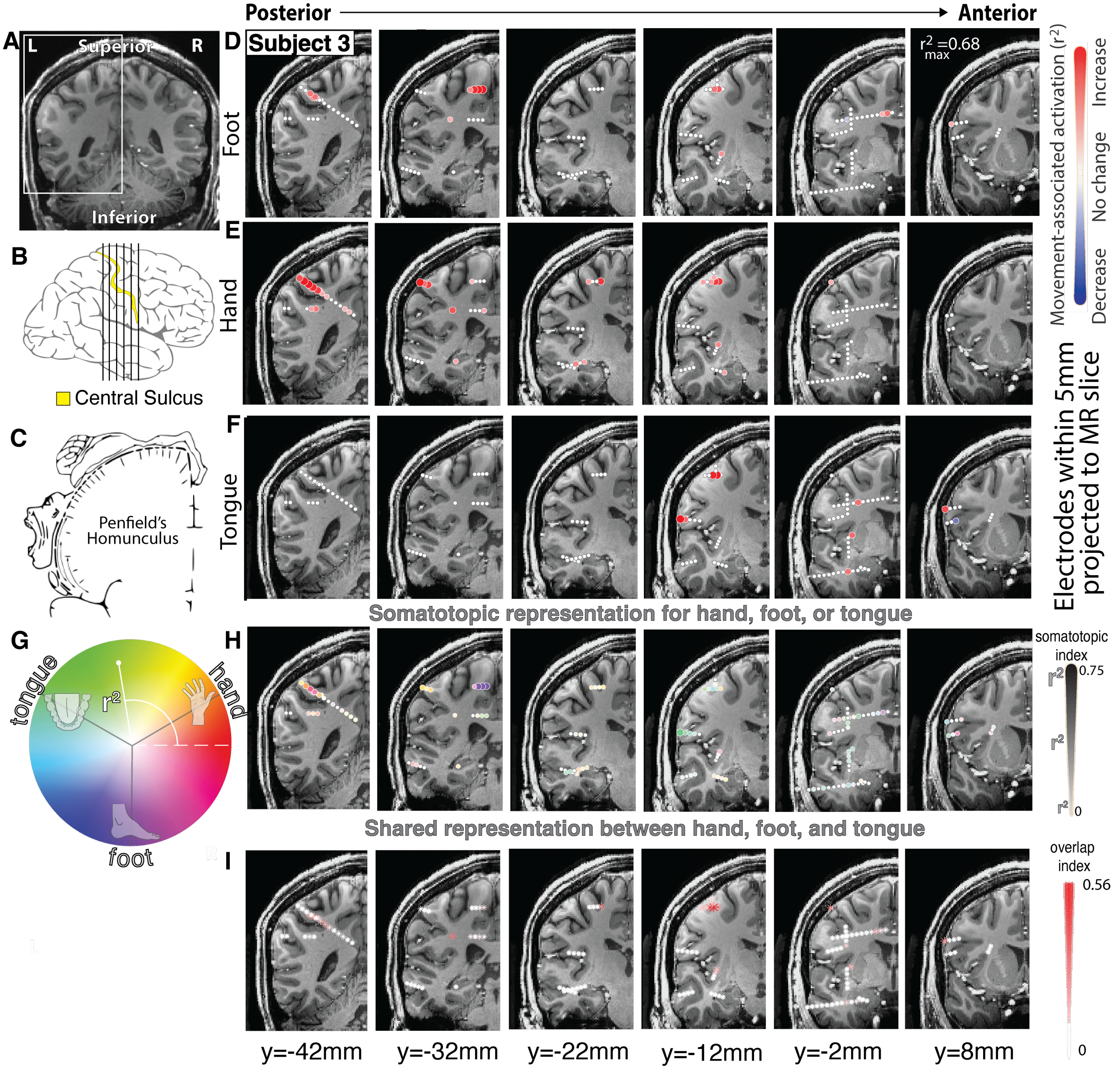

Supplementary Figure 22. Volumetric electrophysiological changes during simple body movements. A. Coronal T1 MRI, with inset box assessed throughout figure. **B.** Coronal slices throughout central sulcus / precentral gyrus shown with black vertical lines. **C.** The classic Penfield motor homunculus from awake stimulation shown as reference[1]. **D-F.** Maps of sEEG power spectral change in the 65-115Hz range during foot, hand, and tongue movement, respectively. Maximum scaling of the color bar is noted in the top right of each row. **G.** Circular colormap showing the color scheme used to indicate somatotopic tuning (H). Color reflects the presence of somatotopic tuning while diameter and intensity indicate the magnitude. Note that a channel that is equally active (even if highly so) during all 3 movement types will be plotted small and white, even if strongly tuned to each movement. **H.** Movement somatotopic maps using scale from (G). **I.** Maps of shared activity in movement (geometric mean of hand, tongue, and foot r^2 values). Insignificant channels are plotted in white in all panels.

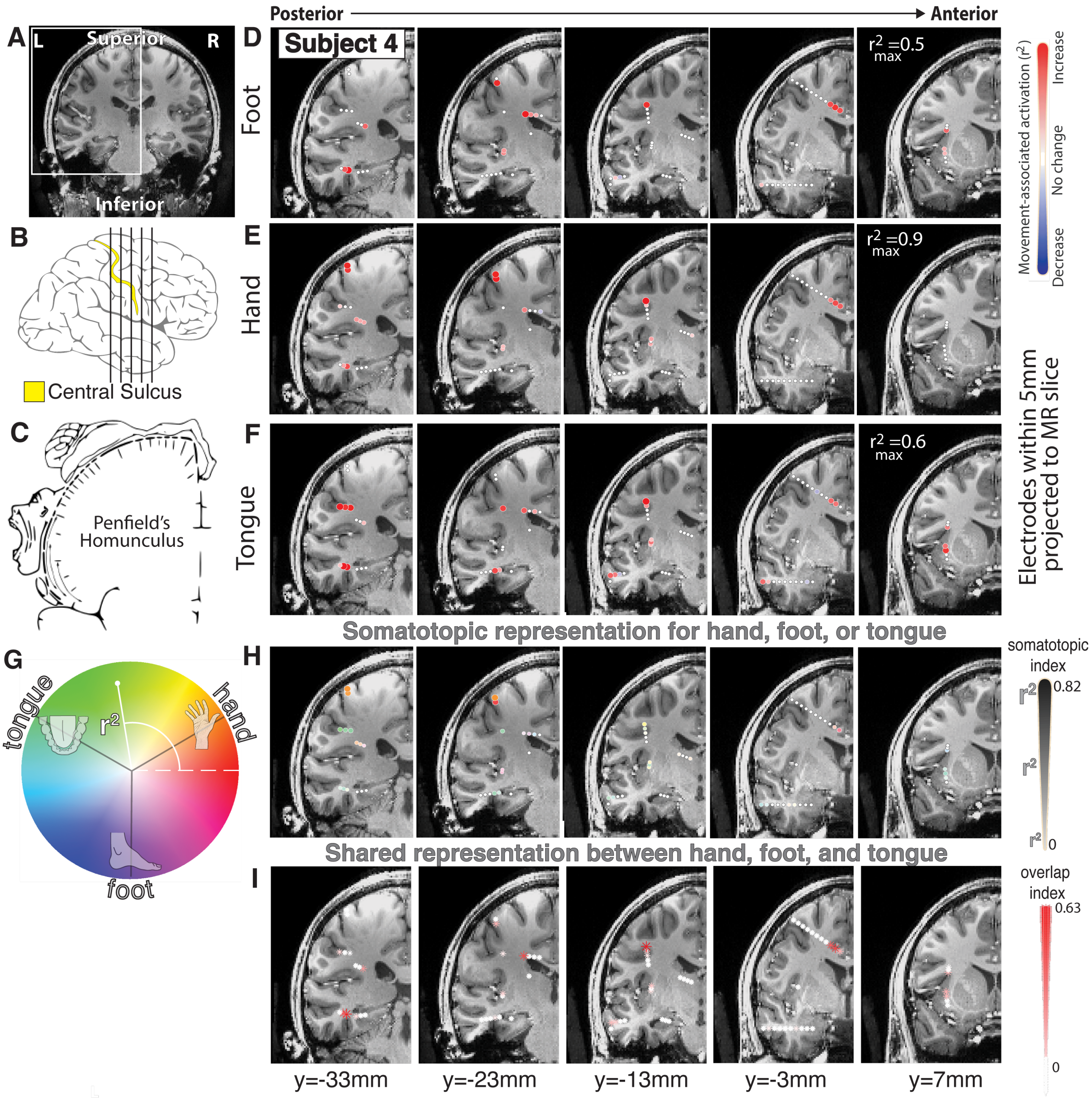

Supplementary Figure 23. Volumetric electrophysiological changes during simple body movements. A. Coronal T1 MRI, with inset box assessed throughout figure. **B.** Coronal slices throughout central sulcus / precentral gyrus shown with black vertical lines. **C.** The classic Penfield motor homunculus from awake stimulation shown as reference[1]. **D-F.** Maps of sEEG power spectral change in the 65-115Hz range during foot, hand, and tongue movement, respectively. Maximum scaling of the color bar is noted in the top right of each row. **G.** Circular colormap showing the color scheme used to indicate somatotopic tuning (H). Color reflects the presence of somatotopic tuning while diameter and intensity indicate the magnitude. Note that a channel that is equally active (even if highly so) during all 3 movement types will be plotted small and white, even if strongly tuned to each movement. **H.** Movement somatotopic maps using scale from (G). **I.** Maps of shared activity in movement (geometric mean of hand, tongue, and foot r^2 values). Insignificant channels are plotted in white in all panels.

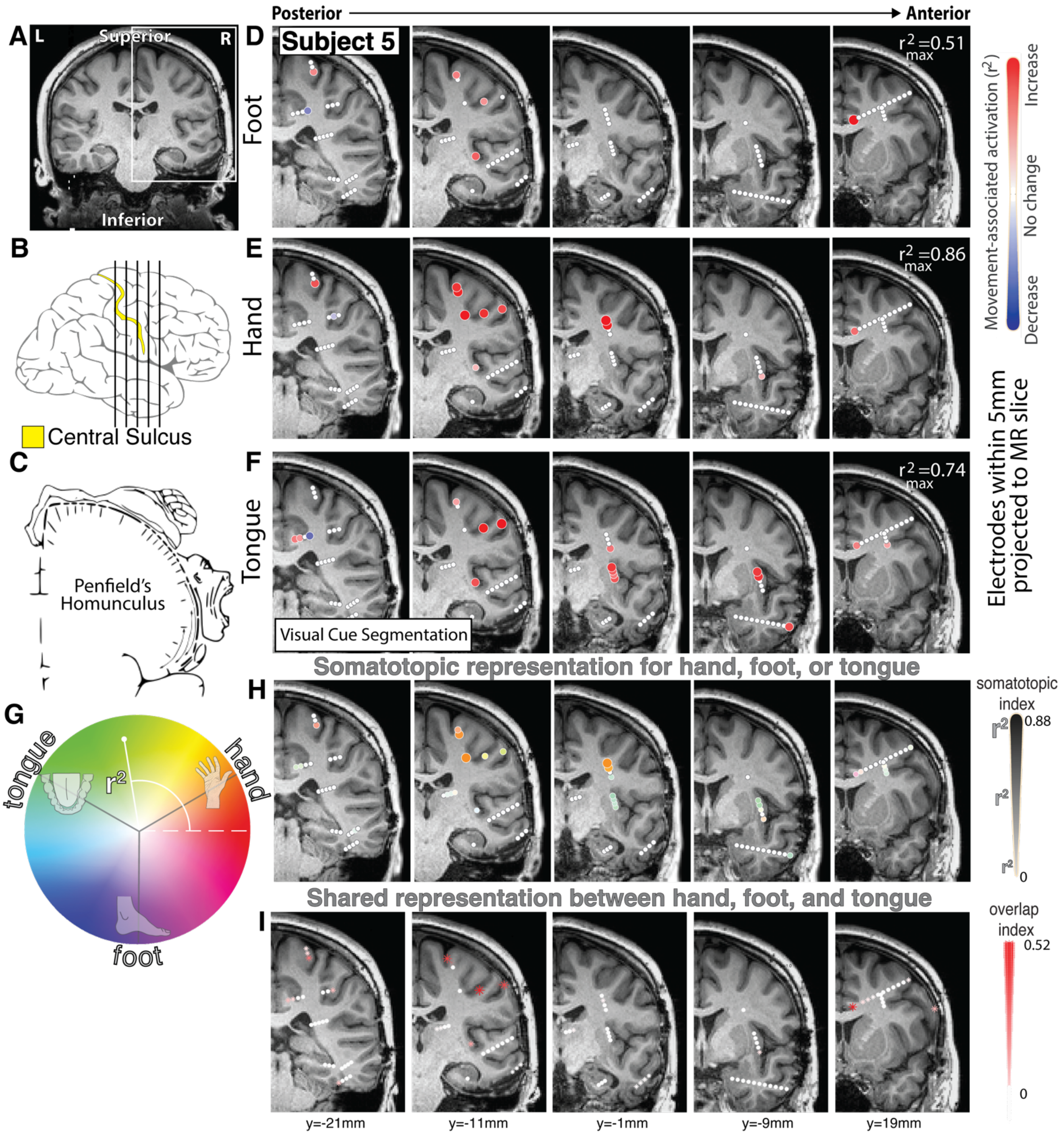

Supplementary Figure 24. Volumetric electrophysiological changes during simple body movements. A. Coronal T1 MRI, with inset box assessed throughout figure. **B.** Coronal slices throughout central sulcus / precentral gyrus shown with black vertical lines. **C.** The classic Penfield motor homunculus from awake stimulation shown as reference[1]. **D-F.** Maps of sEEG power spectral change in the 65-115Hz range during foot, hand, and tongue movement, respectively. Maximum scaling of the color bar is noted in the top right of each row. **G.** Circular colormap showing the color scheme used to indicate somatotopic tuning (H). Color reflects the presence of somatotopic tuning while diameter and intensity indicate the magnitude. Note that a channel that is equally active (even if highly so) during all 3 movement types will be plotted small and white, even if strongly tuned to each movement. **H.** Movement somatotopic maps using scale from (G). **I.** Maps of shared activity in movement (geometric mean of hand, tongue, and foot r^2 values). Insignificant channels are plotted in white in all panels

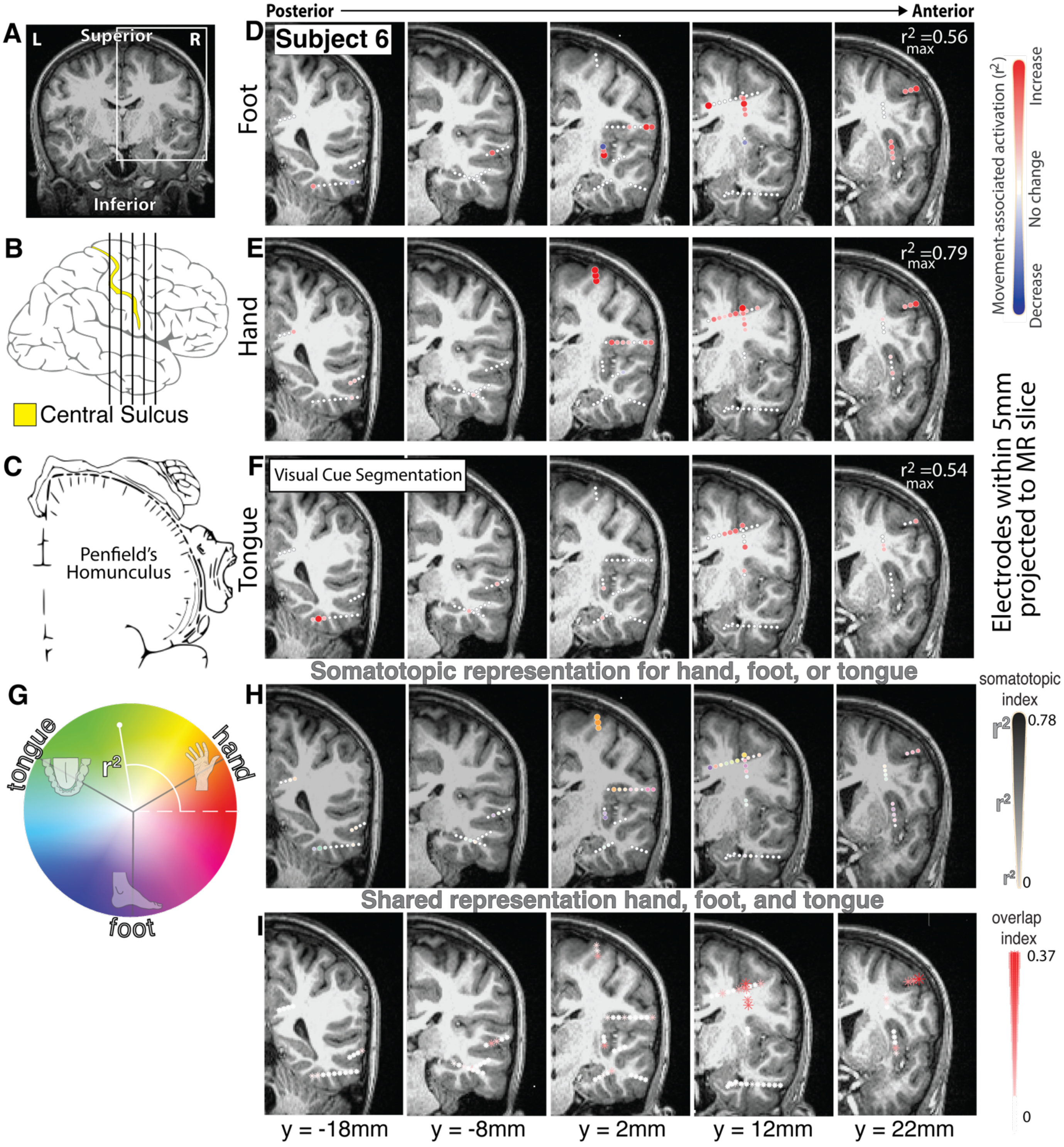

Supplementary Figure 25. Volumetric electrophysiological changes during simple body movements. A. Coronal T1 MRI, with inset box assessed throughout figure. **B.** Coronal slices throughout central sulcus / precentral gyrus shown with black vertical lines. **C.** The classic Penfield motor homunculus from awake stimulation shown as reference[1]. **D-F.** Maps of sEEG power spectral change in the 65-115Hz range during foot, hand, and tongue movement, respectively. Maximum scaling of the color bar is noted in the top right of each row. **G.** Circular colormap showing the color scheme used to indicate somatotopic tuning (H). Color reflects the presence of somatotopic tuning while diameter and intensity indicate the magnitude. Note that a channel that is equally active (even if highly so) during all 3 movement types will be plotted small and white, even if strongly tuned to each movement. **H.** Movement somatotopic maps using scale from (G). **I.** Maps of shared activity in movement (geometric mean of hand, tongue, and foot r^2 values). Insignificant channels are plotted in white in all panels.

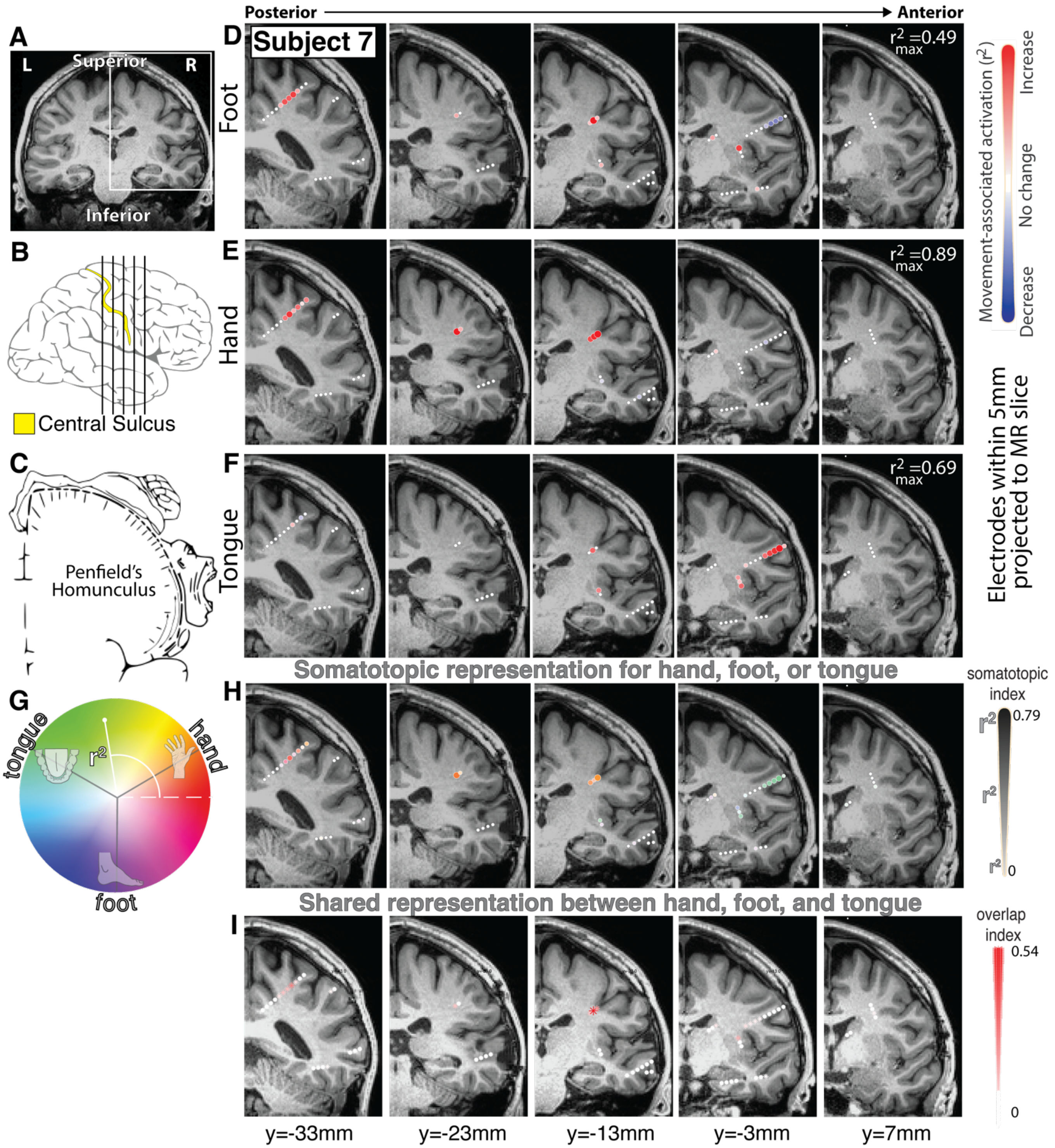

Supplementary Figure 26. Volumetric electrophysiological changes during simple body movements. A. Coronal T1 MRI, with inset box assessed throughout figure. **B.** Coronal slices throughout central sulcus / precentral gyrus shown with black vertical lines. **C.** The classic Penfield motor homunculus from awake stimulation shown as reference[1]. **D-F.** Maps of sEEG power spectral change in the 65-115Hz range during foot, hand, and tongue movement, respectively. Maximum scaling of the color bar is noted in the top right of each row. **G.** Circular colormap showing the color scheme used to indicate somatotopic tuning (H). Color reflects the presence of somatotopic tuning while diameter and intensity indicate the magnitude. Note that a channel that is equally active (even if highly so) during all 3 movement types will be plotted small and white, even if strongly tuned to each movement. **H.** Movement somatotopic maps using scale from (G). **I.** Maps of shared activity in movement (geometric mean of hand, tongue, and foot r^2 values). Insignificant channels are plotted in white in all panels.

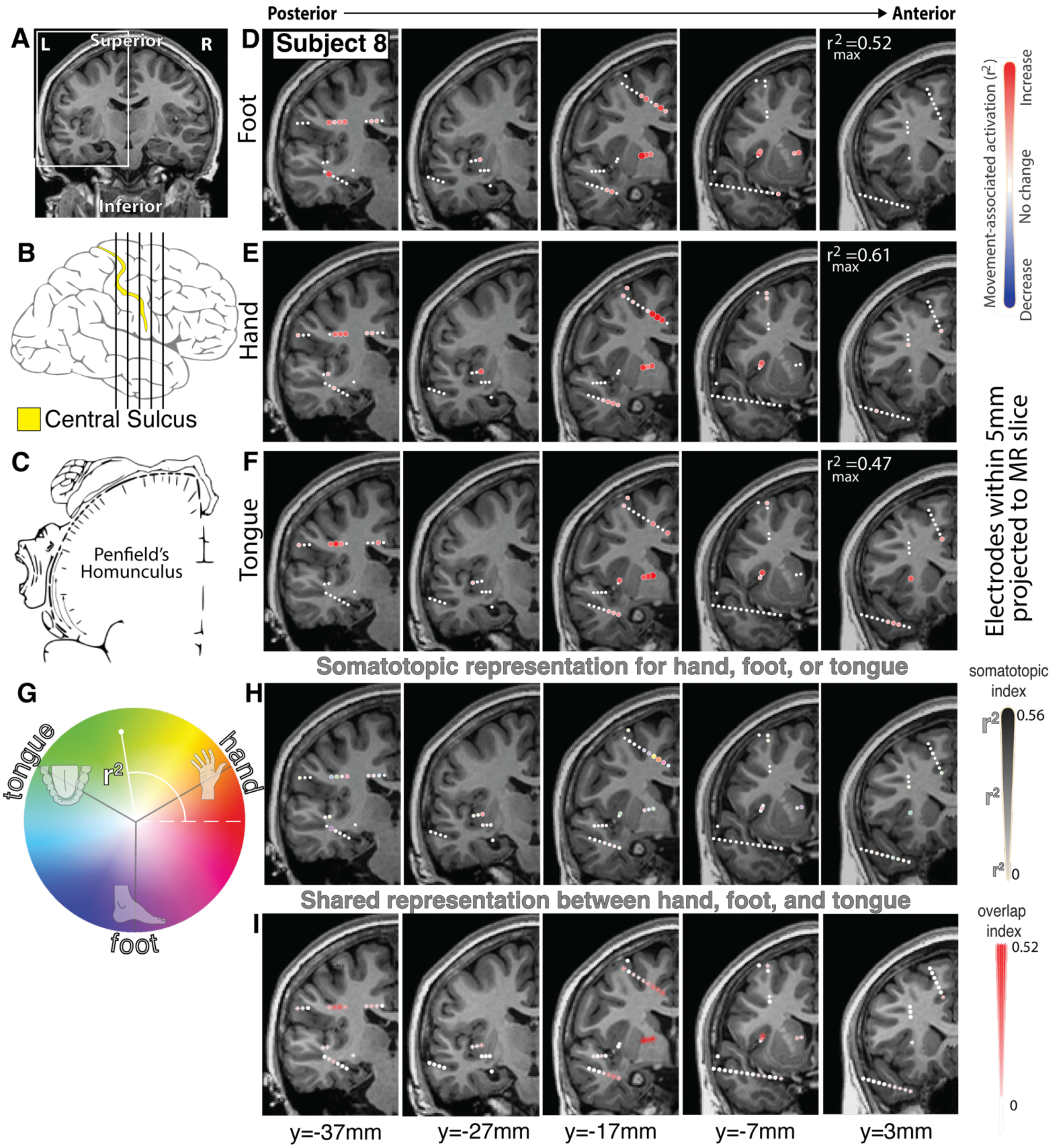

Supplementary Figure 27. Volumetric electrophysiological changes during simple body movements. A. Coronal T1 MRI, with inset box assessed throughout figure. **B.** Coronal slices throughout central sulcus / precentral gyrus shown with black vertical lines. **C.** The classic Penfield motor homunculus from awake stimulation shown as reference[1]. **D-F.** Maps of sEEG power spectral change in the 65-115Hz range during foot, hand, and tongue movement, respectively. Maximum scaling of the color bar is noted in the top right of each row. **G.** Circular colormap showing the color scheme used to indicate somatotopic tuning (H). Color reflects the presence of somatotopic tuning while diameter and intensity indicate the magnitude. Note that a channel that is equally active (even if highly so) during all 3 movement types will be plotted small and white, even if strongly tuned to each movement. **H.** Movement somatotopic maps using scale from (G). **I.** Maps of shared activity in movement (geometric mean of hand, tongue, and foot r^2 values). Insignificant channels are plotted in white in all panels.

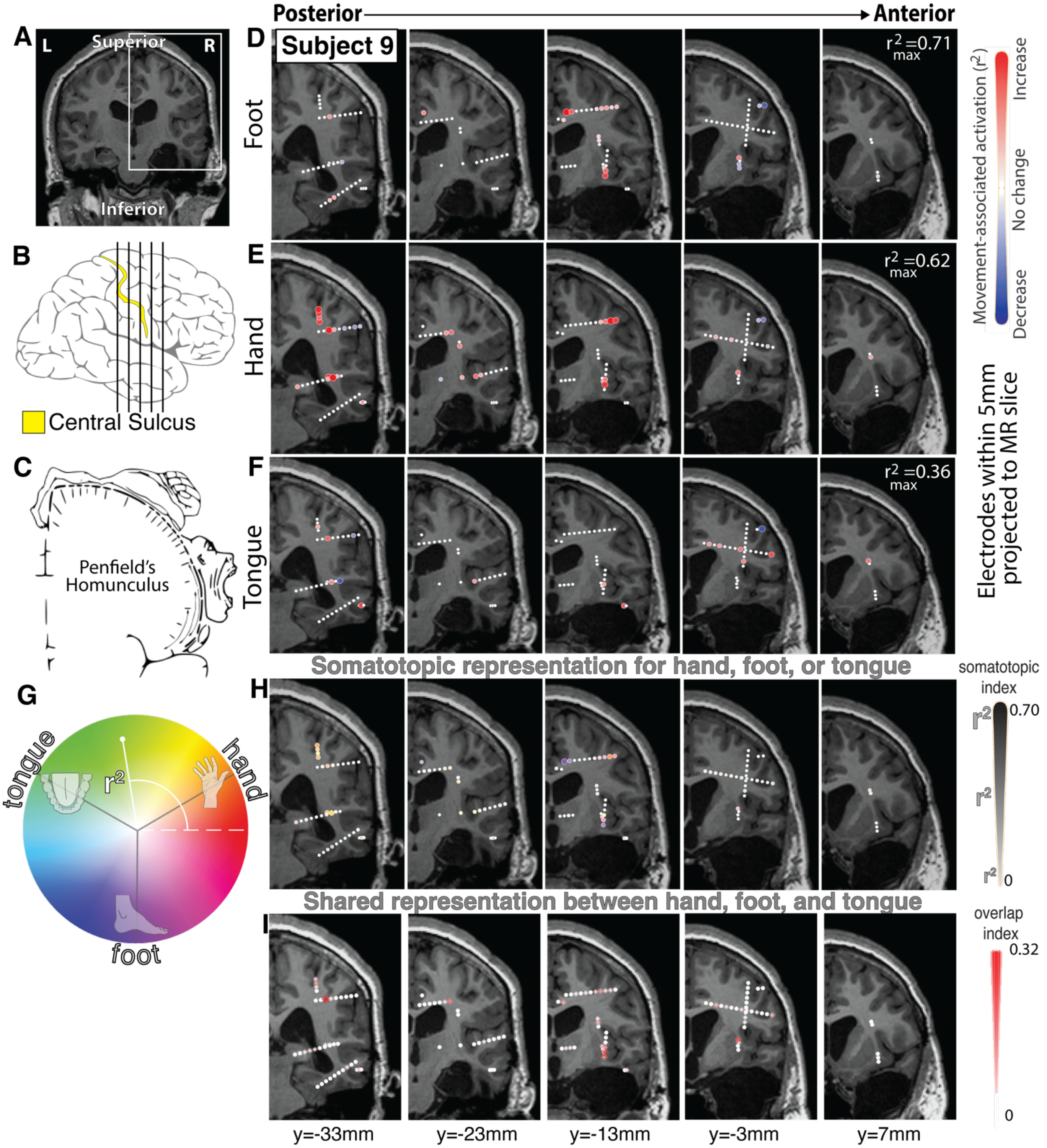

Supplementary Figure 28. Volumetric electrophysiological changes during simple body movements. A. Coronal T1 MRI, with inset box assessed throughout figure. **B.** Coronal slices throughout central sulcus / precentral gyrus shown with black vertical lines. **C.** The classic Penfield motor homunculus from awake stimulation shown as reference[1]. **D-F.** Maps of sEEG power spectral change in the 65-115Hz range during foot, hand, and tongue movement, respectively. Maximum scaling of the color bar is noted in the top right of each row. **G.** Circular colormap showing the color scheme used to indicate somatotopic tuning (H). Color reflects the presence of somatotopic tuning while diameter and intensity indicate the magnitude. Note that a channel that is equally active (even if highly so) during all 3 movement types will be plotted small and white, even if strongly tuned to each movement. **H.** Movement somatotopic maps using scale from (G). **I.** Maps of shared activity in movement (geometric mean of hand, tongue, and foot r^2 values). Insignificant channels are plotted in white in all panels.

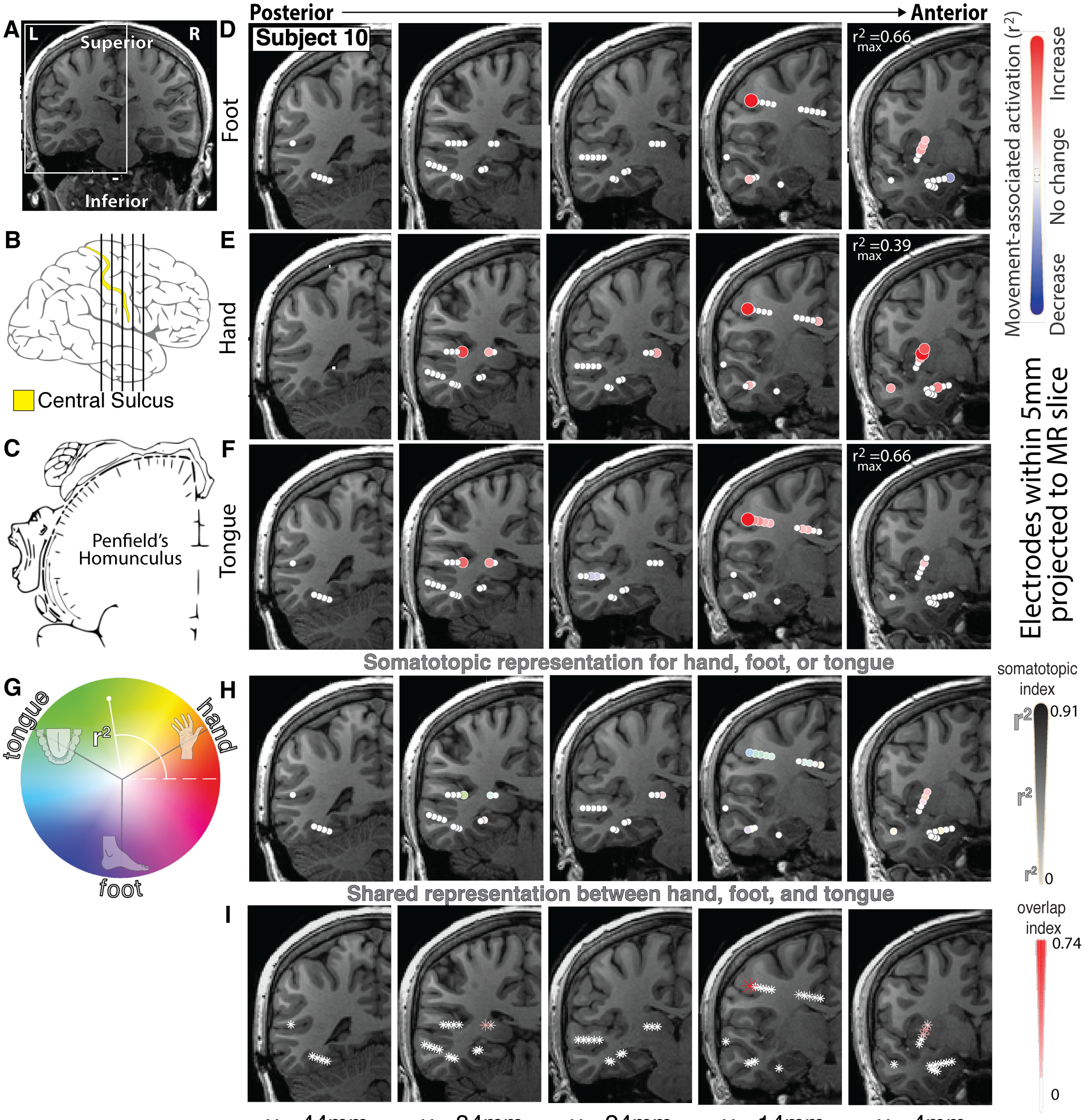

Supplementary Figure 29. Volumetric electrophysiological changes during simple body movements. A. Coronal T1 MRI, with inset box assessed throughout figure. **B.** Coronal slices throughout central sulcus / precentral gyrus shown with black vertical lines. **C.** The classic Penfield motor homunculus from awake stimulation shown as reference[1]. **D-F.** Maps of sEEG power spectral change in the 65-115Hz range during foot, hand, and tongue movement, respectively. Maximum scaling of the color bar is noted in the top right of each row. **G.** Circular colormap showing the color scheme used to indicate somatotopic tuning (H). Color reflects the presence of somatotopic tuning while diameter and intensity indicate the magnitude. Note that a channel that is equally active (even if highly so) during all 3 movement types will be plotted small and white, even if strongly tuned to each movement. **H.** Movement somatotopic maps using scale from (G). **I.** Maps of shared activity in movement (geometric mean of hand, tongue, and foot r^2 values). Insignificant channels are plotted in white in all panels

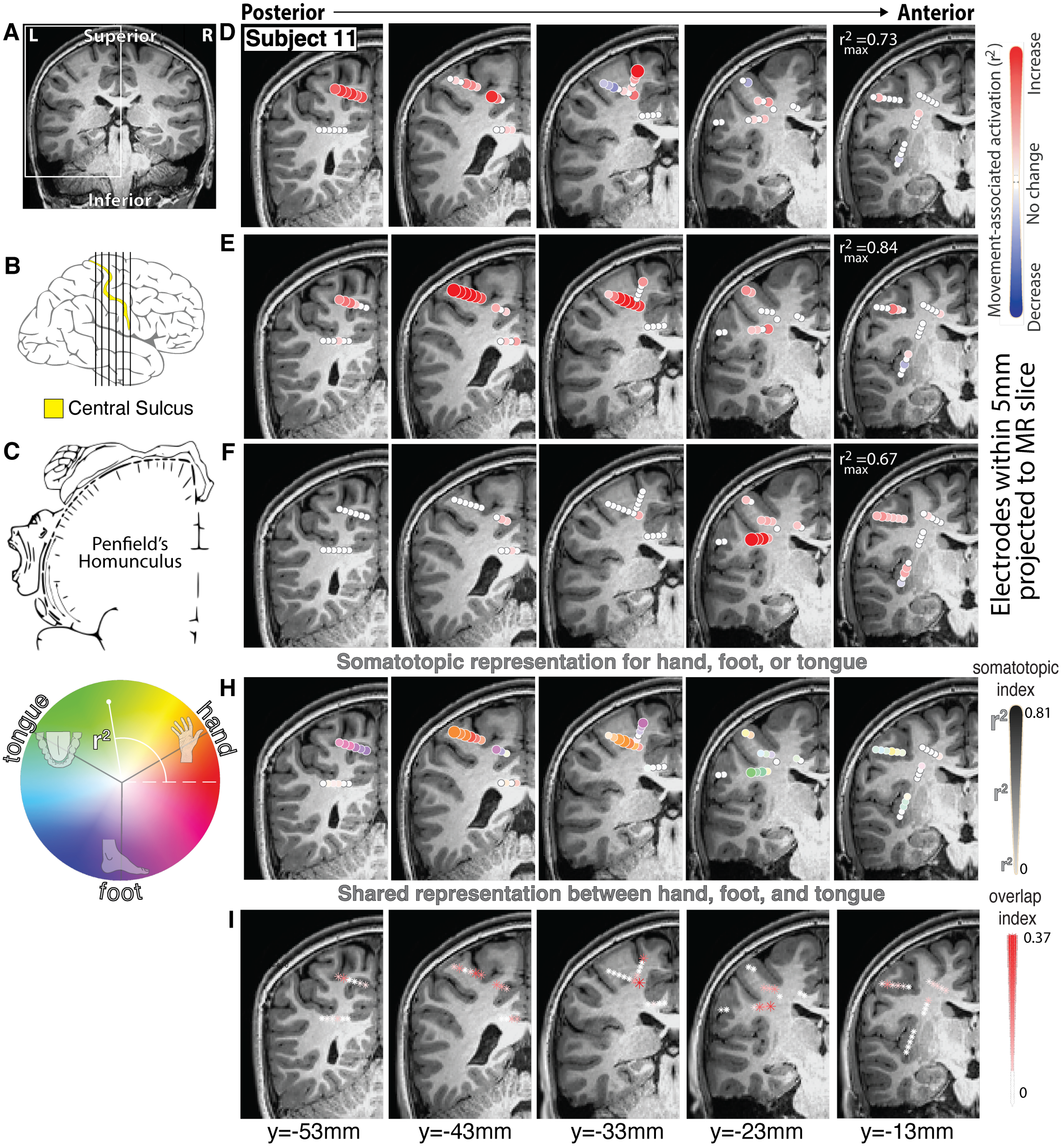

Supplementary Figure 30. Volumetric electrophysiological changes during simple body movements. A. Coronal T1 MRI, with inset box assessed throughout figure. **B.** Coronal slices throughout central sulcus / precentral gyrus shown with black vertical lines. **C.** The classic Penfield motor homunculus from awake stimulation shown as reference[1]. **D-F.** Maps of sEEG power spectral change in the 65-115Hz range during foot, hand, and tongue movement, respectively. Maximum scaling of the color bar is noted in the top right of each row. **G.** Circular colormap showing the color scheme used to indicate somatotopic tuning (H). Color reflects the presence of somatotopic tuning while diameter and intensity indicate the magnitude. Note that a channel that is equally active (even if highly so) during all 3 movement types will be plotted small and white, even if strongly tuned to each movement. **H.** Movement somatotopic maps using scale from (G). **I.** Maps of shared activity in movement (geometric mean of hand, tongue, and foot r^2 values). Insignificant channels are plotted in white in all panels.

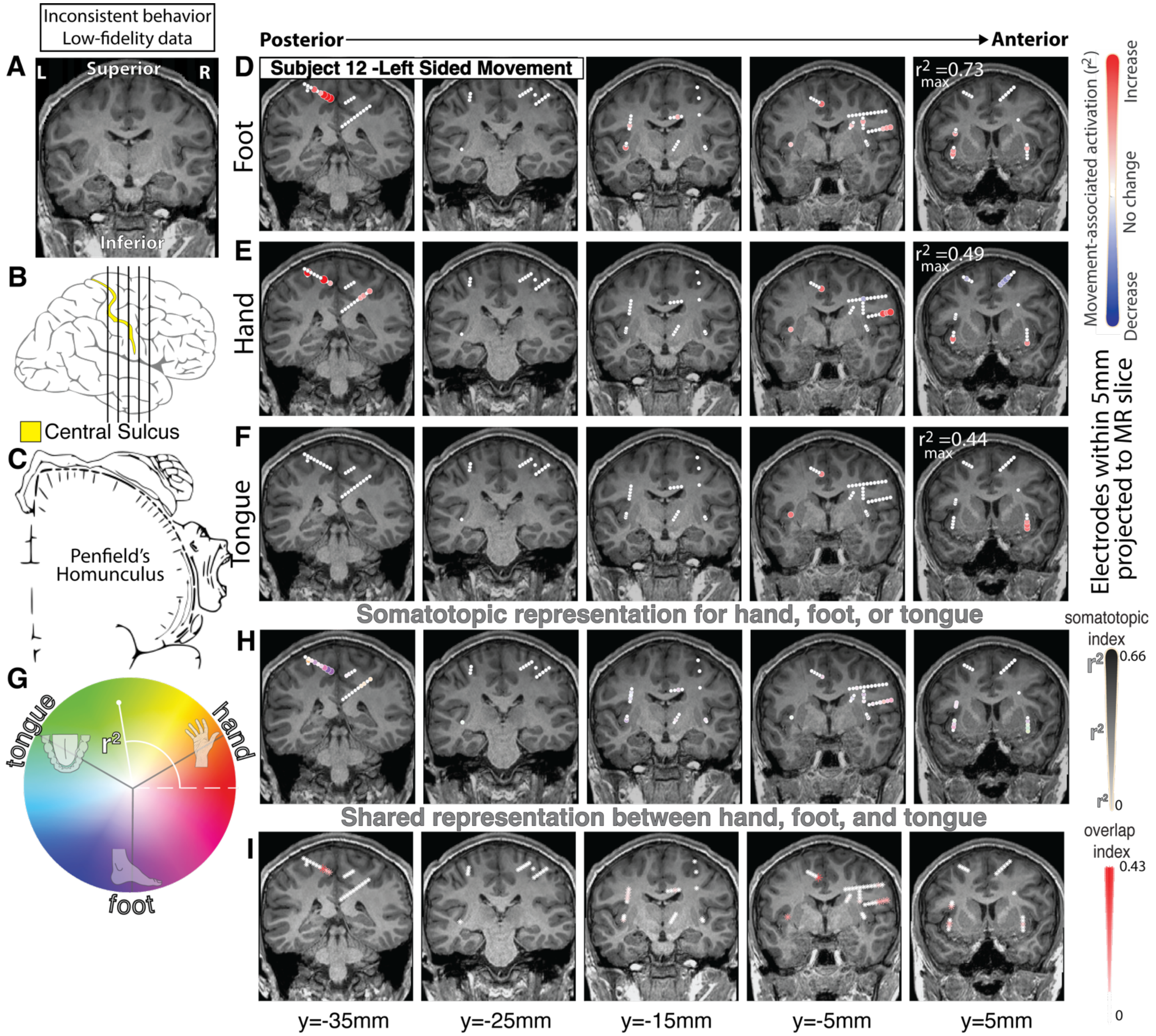

Supplementary Figure 31. Volumetric electrophysiological changes during simple body movements. A. Coronal T1 MRI, with inset box assessed throughout figure. **B.** Coronal slices throughout central sulcus / precentral gyrus shown with black vertical lines. **C.** The classic Penfield motor homunculus from awake stimulation shown as reference[1]. **D-F.** Maps of sEEG power spectral change in the 65-115Hz range during foot, hand, and tongue movement, respectively. Maximum scaling of the color bar is noted in the top right of each row. **G.** Circular colormap showing the color scheme used to indicate somatotopic tuning (H). Color reflects the presence of somatotopic tuning while diameter and intensity indicate the magnitude. Note that a channel that is equally active (even if highly so) during all 3 movement types will be plotted small and white, even if strongly tuned to each movement. **H.** Movement somatotopic maps using scale from (G). **I.** Maps of shared activity in movement (geometric mean of hand, tongue, and foot r^2 values). Insignificant channels are plotted in white in all panels.

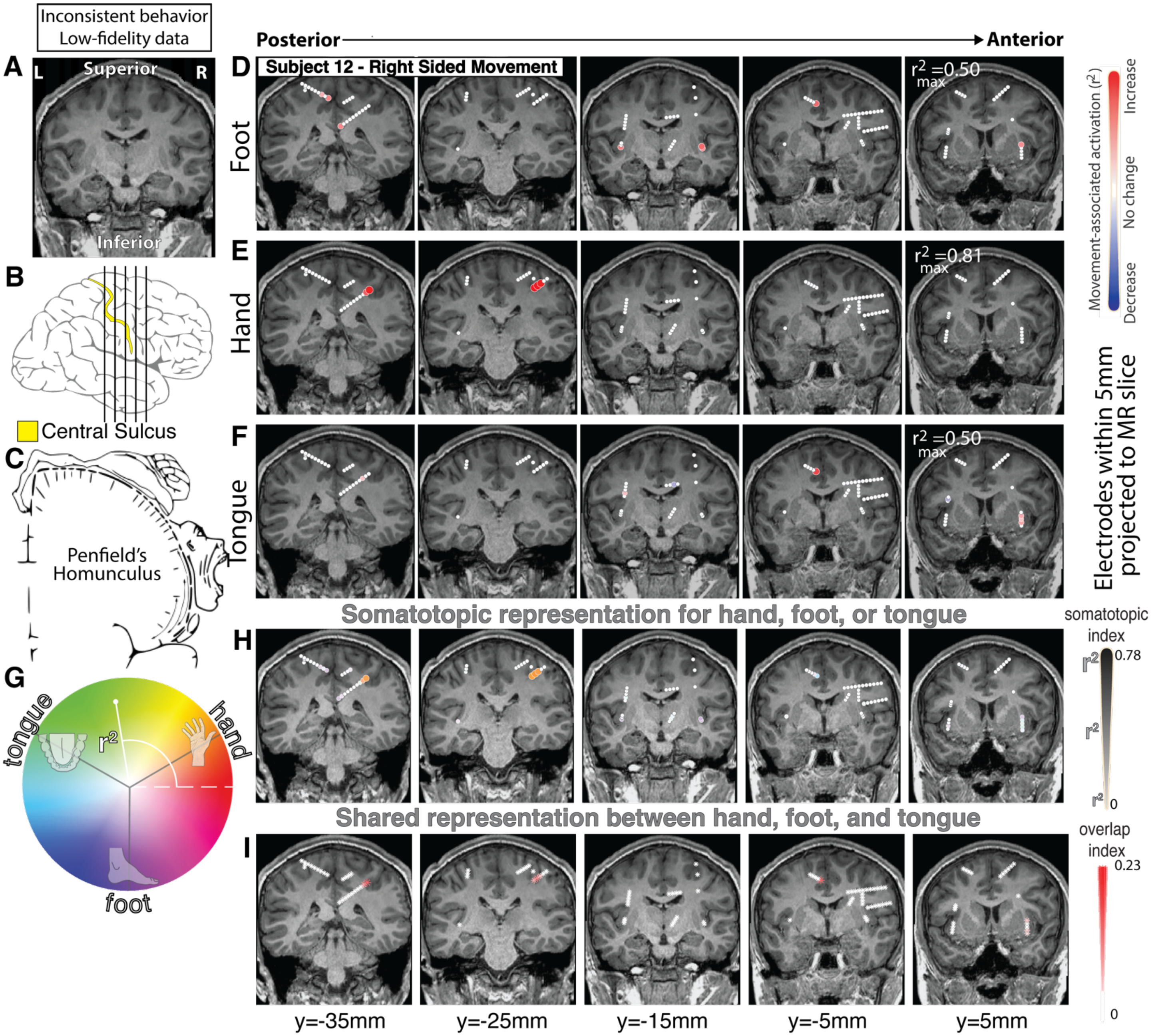

Supplementary Figure 32. Volumetric electrophysiological changes during simple body movements. A. Coronal T1 MRI, with inset box assessed throughout figure. **B.** Coronal slices throughout central sulcus / precentral gyrus shown with black vertical lines. **C.** The classic Penfield motor homunculus from awake stimulation shown as reference[1]. **D-F.** Maps of sEEG power spectral change in the 65-115Hz range during foot, hand, and tongue movement, respectively. Maximum scaling of the color bar is noted in the top right of each row. **G.** Circular colormap showing the color scheme used to indicate somatotopic tuning (H). Color reflects the presence of somatotopic tuning while diameter and intensity indicate the magnitude. Note that a channel that is equally active (even if highly so) during all 3 movement types will be plotted small and white, even if strongly tuned to each movement. **H.** Movement somatotopic maps using scale from (G). **I.** Maps of shared activity in movement (geometric mean of hand, tongue, and foot r^2 values). Insignificant channels are plotted in white in all panels.

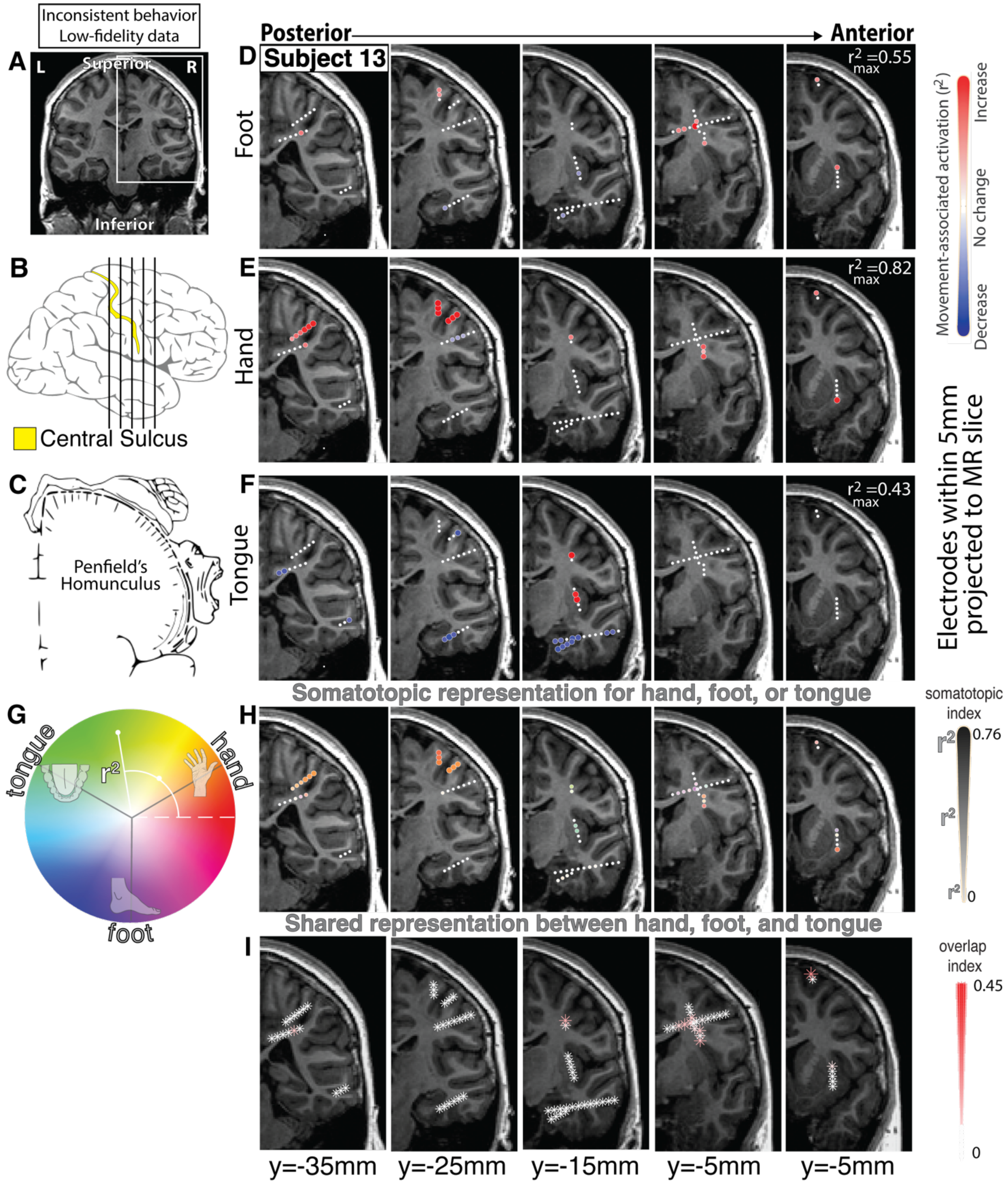

Supplementary Figure 33. Volumetric electrophysiological changes during simple body movements. A. Coronal T1 MRI, with inset box assessed throughout figure. **B.** Coronal slices throughout central sulcus / precentral gyrus shown with black vertical lines. **C.** The classic Penfield motor homunculus from awake stimulation shown as reference[1]. **D-F.** Maps of sEEG power spectral change in the 65-115Hz range during foot, hand, and tongue movement, respectively. Maximum scaling of the color bar is noted in the top right of each row. **G.** Circular colormap showing the color scheme used to indicate somatotopic tuning (H). Color reflects the presence of somatotopic tuning while diameter and intensity indicate the magnitude. Note that a channel that is equally active (even if highly so) during all 3 movement types will be plotted small and white, even if strongly tuned to each movement. **H.** Movement somatotopic maps using scale from (G). **I.** Maps of shared activity in movement (geometric mean of hand, tongue, and foot r^2 values). Insignificant channels are plotted in white in all panels.
