# Supplementary material for: A motor association area in the depths of the central sulcus
Source: Nat Neurosci. 2023 May 18;26(7):1165–9. doi: 10.1038/s41593-023-01346-z (PMC10322697; doi:10.1038/s41593-023-01346-z)
Supplement: Supplementary file 2 — Reporting Summary [file 41593_2023_1346_MOESM2_ESM.pdf]

nature portfolio

Corresponding author(s): Kai Miller
Michael Jensen

Last updated by author(s): Apr 14, 2022

Reporting Summary

Nature Portfolio wishes to improve the reproducibility of the work that we publish. This form provides structure for consistency and transparency in reporting. For further information on Nature Portfolio policies, see our Editorial Policies and the Editorial Policy Checklist.

Statistics

For all statistical analyses, confirm that the following items are present in the figure legend, table legend, main text, or Methods section.

n/a	Confirmed	
☐	☒	The exact sample size (*n*) for each experimental group/condition, given as a discrete number and unit of measurement
☒	☐	A statement on whether measurements were taken from distinct samples or whether the same sample was measured repeatedly
☐	☒	The statistical test(s) used AND whether they are one- or two-sided<br>*Only common tests should be described solely by name; describe more complex techniques in the Methods section.*
☒	☐	A description of all covariates tested
☐	☒	A description of any assumptions or corrections, such as tests of normality and adjustment for multiple comparisons
☒	☐	A full description of the statistical parameters including central tendency (e.g. means) or other basic estimates (e.g. regression coefficient) AND variation (e.g. standard deviation) or associated estimates of uncertainty (e.g. confidence intervals)
☐	☒	For null hypothesis testing, the test statistic (e.g. *F*, *t*, *r*) with confidence intervals, effect sizes, degrees of freedom and *P* value noted<br>*Give P values as exact values whenever suitable.*
☒	☐	For Bayesian analysis, information on the choice of priors and Markov chain Monte Carlo settings
☒	☐	For hierarchical and complex designs, identification of the appropriate level for tests and full reporting of outcomes
☐	☒	Estimates of effect sizes (e.g. Cohen's *d*, Pearson's *r*), indicating how they were calculated

Our web collection on statistics for biologists contains articles on many of the points above.

Software and code

Policy information about availability of computer code

Data collection	Our data and analysis code are publicly available on the OSF data repository at the following link: https://osf.io/p5n2k.<br>Matlab versions 2022a (M.A.J), 2020a (K.J.M) were used data analyses, and version 2022a (G.O.V) were used for data curation. SPM12 r7771 were across all data curation.
Data analysis	Our data were analyzed in MATLAB using code we wrote that is available at: https://osf.io/p5n2k

For manuscripts utilizing custom algorithms or software that are central to the research but not yet described in published literature, software must be made available to editors and reviewers. We strongly encourage code deposition in a community repository (e.g. GitHub). See the Nature Portfolio guidelines for submitting code & software for further information.

Data

Policy information about availability of data

All manuscripts must include a data availability statement. This statement should provide the following information, where applicable:

- Accession codes, unique identifiers, or web links for publicly available datasets
- A description of any restrictions on data availability
- For clinical datasets or third party data, please ensure that the statement adheres to our policy

Our data are publicly available on the OSF data repository at the following link: https://osf.io/p5n2k
No data were excluded from this set and all data are anonymized. A README document is found in the link, above which includes a detailed description of all

variables included in the data files.
These data adhere to the policy attached.

Human research participants

Policy information about studies involving human research participants and Sex and Gender in Research.

Reporting on sex and gender	All patients included in the study were given the opportunity to refuse participation in research. No selection was made based on sex or gender.
Population characteristics	Thirteen patients (6 females, 11-20 years of age) participated in our study, each of whom underwent placement of 10-15 sEEG electrode leads for seizure network characterization in the treatment of drug resistant partial epilepsy.
Recruitment	Patients of the senior author were approached by a non-clinical staff member and asked if they wish to participate in the study. As the senior author is a pediatric neurosurgeon and his patients contribute to our study group (Supplemental Table 1), subjects are a younger distribution than the general population demographic. All potential subjects in the 15 month study period were included, eliminating the possibility of experimenter bias.
Ethics oversight	The study was conducted according to the guidelines of the Declaration of Helsinki and approved by the Institutional Review Board of the Mayo Clinic IRB# 15-006530, which also authorizes sharing of the data. Each patient / representative voluntarily provided independent written informed consent to participate in this study as specifically described in the IRB review (with the consent form independently approved by the IRB).

Note that full information on the approval of the study protocol must also be provided in the manuscript.

Field-specific reporting

Please select the one below that is the best fit for your research. If you are not sure, read the appropriate sections before making your selection.

☒ Life sciences ☐ Behavioural & social sciences ☐ Ecological, evolutionary & environmental sciences

For a reference copy of the document with all sections, see nature.com/documents/nr-reporting-summary-flat.pdf

Life sciences study design

All studies must disclose on these points even when the disclosure is negative.

Sample size	Our sample size was 13 human subjects (patients). All subjects who consented to participation were recruited consecutively for 15 months until the sample size was similar to previous studies (references 3, 15).
Data exclusions	No data were excluded from the study
Replication	Findings were replicated in 13 consecutive patients.
Randomization	No different experimental conditions were applied to the subjects and randomization was not possible.
Blinding	Data collection and analysis were not performed blind to the conditions of the experiments.

Reporting for specific materials, systems and methods

We require information from authors about some types of materials, experimental systems and methods used in many studies. Here, indicate whether each material, system or method listed is relevant to your study. If you are not sure if a list item applies to your research, read the appropriate section before selecting a response.

Materials & experimental systems

n/a	Involved in the study
☒	☐ Antibodies
☒	☐ Eukaryotic cell lines
☒	☐ Palaeontology and archaeology
☒	☐ Animals and other organisms
☒	☐ Clinical data
☒	☐ Dual use research of concern

Methods

n/a	Involved in the study
☒	☐ ChIP-seq
☒	☐ Flow cytometry
☒	☐ MRI-based neuroimaging
